# mbDenoise: microbiome data denoising using zero-inflated probabilistic principal components analysis

**DOI:** 10.1186/s13059-022-02657-3

**Published:** 2022-04-14

**Authors:** Yanyan Zeng, Jing Li, Chaochun Wei, Hongyu Zhao, Wang Tao

**Affiliations:** 1 Department of Bioinformatics and Biostatistics, Shanghai Jiao Tong University, Shanghai, China; 2 Department of Biostatistics, Yale University, New Haven, CT USA; 3 SJTU-Yale Joint Center for Biostatistics and Data Science, Shanghai Jiao Tong University, Shanghai, China; 4 Department of Statistics, School of Mathematical Sciences, Shanghai Jiao Tong University, Shanghai, China; 5 Joint International Research Laboratory of Metabolic & Developmental Sciences, Shanghai Jiao Tong University, Shanghai, China

**Keywords:** Biological zeros, Differential abundance, Diversity, Negative binomial, Normalization

## Abstract

The analysis of microbiome data has several technical challenges. In particular, count matrices contain a large proportion of zeros, some of which are biological, whereas others are technical. Furthermore, the measurements suffer from unequal sequencing depth, overdispersion, and data redundancy. These nuisance factors introduce substantial noise. We propose an accurate and robust method, mbDenoise, for denoising microbiome data. Assuming a zero-inflated probabilistic PCA (ZIPPCA) model, mbDenoise uses variational approximation to learn the latent structure and recovers the true abundance levels using the posterior, borrowing information across samples and taxa. mbDenoise outperforms state-of-the-art methods to extract the signal for downstream analyses.

**Supplementary Information:**

The online version contains supplementary material available at (10.1186/s13059-022-02657-3).

## Background

Advances in DNA sequencing technologies have revolutionized the study of microbial communities in many diverse environments, and in particular have enabled researchers to better understand the implications of microbiome variation in human health and disease. These developments have led to a rapidly growing number of microbiome studies and unprecedented volumes of sequencing count data. Despite improvements in experimental methods and protocols, the analysis and interpretation of these data are complicated by nuisance factors such as uneven sequencing depth, overdispersion, data redundancy, and especially data sparsity [1]. These characteristics lead to substantial noise in microbiome data, making it difficult to distinguish between technical and biological variation, and thus, if not addressed, can obstruct high-level analyses, such as unconstrained ordination, alpha and beta diversity calculation, and differential abundance testing [2].

First, the total number of reads per sample (observed sequencing depth or library size) is strongly affected by the sequencing platform used and the number of samples that are multiplexed per run, and can vary by orders of magnitude across samples. Consequently, unequal sequencing depth represents the deficiency of the sequencing process instead of the real biological variation. Second, sampling is another obvious source of technical variation due to limited sequencing depth, and it is known that there is overdispersion in sequencing data, which refers to the fact that read counts are more variable than what is expected according to a Poisson distribution. These variations can usually be accounted for using a discrete probability model, and many existing approaches use the negative binomial distribution as a means of controlling for overdispersion [3, 4]. Third, the microbiome is functionally redundant, that is, some taxa perform similar functions in communities and ecosystems, and redundant taxa may therefore be substitutable with little impact on ecosystem processes [5]. As a result of the correlations between microbes, the intrinsic dimension of abundance data is typically smaller than the ambient dimension of feature space. This data redundancy can be addressed by a low-rank approximation [6], which can potentially eliminate the problem of overfitting and improve prediction accuracy, especially when the sample-size to feature-dimension ratio is small.

Microbiome data are often extremely sparse, that is, the count matrices contain a large proportion of zero values. This sparsity can arise for two reasons. First, microbes are present in the environment but not detected due to low sequencing depth and sampling variation. We refer to these zeros as technical zeros. Second, it is possible that some microbes are incapable of living in the environment and truly never represented. It could also be that an intrinsic stochasticity in the biochemical process inhibits our ability to detect these microbes [7]. We call the resulting zeros biological zeros. For accurate analysis of microbiome data, biological signal should be separated from technical noise, and choosing a method that adequately addresses variability in sequencing depth, data sparsity and overdispersion, and data redundancy has been the subject of active research.

A line of study distinguishes technical zeros from biological zeros, and replaces or imputes technical zeros by nonzero values. Jiang et al. [8] proposed the first method, mbImpute, for microbiome data. It is a two-stage procedure. First, taxa abundance values are fitted by a gamma-normal mixture model, and those that need imputation are identified. Second, data imputation is performed by penalized linear regressions that combines the predictive power of similar taxa, similar samples, and sample covariates. A related approach, called scImpute [9], was developed specifically for single-cell RNA-seq (scRNA-seq) data. Expression values of genes in a cell affected by dropout events are determined by the gamma-normal mixture model, and they are imputed using non-negative least squares that borrows information of the same gene in other similar cells. These two methods divide every count in a sample by the sequencing depth of that sample, and log-transform the relative abundance data. Another method for use on scRNA-seq data, ALRA [10], computes a low-rank approximation by singular vector decomposition to recover true nonzero expression values, and selectively preserves biological zeros at zero expression levels. It utilizes the non-negativity and redundancy of expression matrices, and is motivated by the observation that the nonzero values incorrectly assigned to biological zeros are symmetrically distributed around zero. A major drawback of the above methods is that a threshold needs to be specified so as to decide which zeros do not require imputation.

A different thread directly extracts biological signal buried in technical noise. Huang et al. [11] proposed SAVER to restore scRNA-seq expression data. SAVER assumes a Poisson-gamma mixture model for unique molecule index-based counts, estimates the parameters using penalized Poisson regressions that take advantage of gene-to-gene relationships, and then uses posterior means to recover the expression level of each gene in each cell. A size factor is included in the Poisson model to account for differences in sequencing depth across cells. Unlike scImpute and ALRA, however, SAVER treats all zeros equally, and the authors recommend removing extremely low-abundance genes at the beginning. To account for the distinction between technical and biological zeros, Eraslan et al. [12] proposed a deep learning based autoencoder, DCA, to remove technical noise while retaining biological variation in scRNA-seq data. DCA carries out a likelihood ratio test between the negative binomial (NB) and zero-inflated negative binomial (ZINB) to specify the noise model. The inferred mean matrix of the negative binomial component represents reconstructed gene expression values. Another deep learning method, known as scVI [13], also makes use of autoencoders with a ZINB distribution for embedding scRNA-seq data. scVI assumes a fully Bayesian model and explicitly corrects sequencing depth and batch effect biases. It uses variational inference to approximate the distributions that underlie observed expression values. The key advantages of DCA and scVI are their flexibility and scalability, namely, they can capture nonlinear gene-gene dependencies and scale almost linearly with the number of cells. Although deep learning methods are popular in recent years for analyzing scRNA-seq data, they may not provide useful solutions in the analysis of microbiome data. This is because neural networks have many hidden units and layers that make them prone to overfitting in problems that involve substantial amount of noise and limited data. Unfortunately, the number of samples in microbiome studies is usually in the order of tens or hundreds and much smaller than that of cells in scRNA-seq datasets. The extraction of biologically meaningful information from microbiome data thus requires the development of specialized denoising methods.

**Fig. 1:**
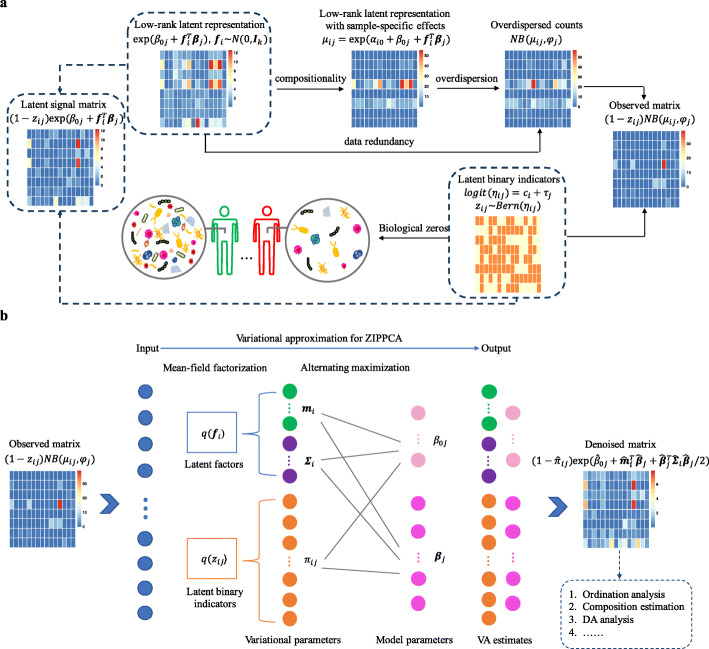
Overview of mbDenoise and the noise model. **a** mbDenoise distinguishes biological zeros from technical zeros, and assumes that the true nonzero abundance data lie on a low-dimensional latent space embedded in the high-dimensional feature space, reflecting the observed redundancy in microbiome data. mbDenoise recovers the true abundance levels, that is, the latent signal matrix, by fitting a zero-inflated probabilistic PCA (ZIPPCA) model. The ZIPPCA framework takes into account uneven library size, overdispersion, and sparsity using a mixture model that consists of a negative binomial count distribution and a point mass at zero. **b** Input data (that is, observed count matrix) are assumed to be samples from this mixture model, and the posterior mean estimate of the latent signal matrix by variational approximation represents the denoised output. The denoised abundance can be used for multiple downstream analysis tasks

We develop mbDenoise, a latent variable modeling approach for denoising microbiome data. mbDenoise borrows information across samples and taxa to decouple biological signal from technical variation. mbDenoise is based on a noise model that extends probabilistic PCA to address the nuisance factors in microbiome data (Fig. 1). The observed count of a taxon in a sample is generated from a ZINB model. The NB component accounts for the presence of overdispersion in count data, and the second component, a point mass at zero, deals with the data sparsity problem and distinguishes between technical and biological zeros. Unobserved sample-specific effects are included in the linear predictor of the NB component to remove technical bias due to differences in sequencing depth. The low-rank representation, that is, the linear combinations of latent factors in the linear predictor, takes advantage of the redundancy in microbiome data and reflects the remaining variation. We call the generative model for mbDenoise zero-inflated probabilistic PCA (ZIPPCA). Environmental variables, if available, can be easily adjusted for in this framework. mbDenoise denoises microbiome data by learning the latent features and then recovering the true abundance levels using the posterior mean. See the “Methods” section for details. Using simulated and real datasets, we extensively investigate the performance of mbDenoise by carrying out downstream statistical analyses on the denoised data, including dimension reduction and ordination analysis, alpha and beta diversity analysis, and differential abundance analysis. We also compare mbDenoise to mbImpute and other state-of-the-art methods.

## Results

### Simulation experiments

We used six simulated examples (M1-M6), each with two different combinations of sample size *n* and number of taxa *p*, to examine the performance of mbDenoise. Below is a brief description of each example. More detailed information can be seen in Additional file 1: Table S1.1.

In example M1, data were generated from the zero-inflated negative binomial model in the “Methods” section. Examples M2 and M3 replaced negative binomial by Poisson and logistic normal multinomial distributions, respectively. Example M4 assumed a zero-inflated log normal distribution, whose positive part generated continuous data instead of counts. These models are extensions of probabilistic PCA or factor analysis models and belong to the general class of generalized latent variable models.

In addition to zero-inflated models, data in example M5, borrowed from Niku et al. [14], were generated from a negative binomial latent variable model, where latent variables followed a mixture of Gaussians, rather than a standard normal distribution as in M1-M4. Example M6 adopted the simulation setting of Cao et al. [15], in which data were drawn from a multinomial distribution with nonrandom factors.

To illustrate how our method can be used in finding overall patterns in microbiome variation and detecting differentially abundant (DA) taxa in the comparison groups of interest, we first assessed the accuracy of estimation and prediction. Then, we examined the performance in terms of composition estimation. Finally, we evaluated the effectiveness of data recovery and its impact on DA analysis. In each case, examples were chosen from M1–M6, and in each example, both *n*<*p* and *n*>*p* were considered, and the results were averaged over 100 data replications. For ease of exposition, results for settings with *n*>*p* were put in the supplementary.

The above simulation scenarios are all model-based, and there may be concerns about the extent to which such set-ups capture the structure of real microbiome data. Some recently developed simulation approaches, such as SparseDOSSA 2 [16], may generate more realistic data. We also applied sparseDOSSA 2 to simulate data for DA analysis. We used the built-in vaginal samples as real source data for sparseDOSSA 2, and set the sample size for each group *n*=100 and the number of taxa *p* was by default 109. The fraction of DA features was 40%. Four values of the effect size (1, 2, 5, and 10) were explored.

### mbDenoise ensures the accuracy of estimation and prediction

To gain preliminary insight into the operating characteristics of mbDenoise, we used simulated examples M1–M5 to evaluate its performance in terms of both estimation of unknown parameters and prediction of latent factors, using two criteria for measuring the dissimilarity between the true and estimated or predicted values: the symmetric Procrustes error [17] and the orthogonal projection distance [18]. For comparison purpose, we included the results of existing methods. These methods can be roughly divided into two categories: (1) algorithm-based methods including PCA, which is linear, and t-distributed stochastic neighbor embedding (t-SNE) [19], which is nonlinear and popular in the machine learning community; and (2) latent variable model-based methods, including negative binomial probabilistic PCA (PPCA-NB) [14, 20], zero-inflated factor analysis (ZIFA) [21], and our method mbDenoise-zinb and its variant mbDenoise-zip. We note that inputs were log2(1+counts) for PCA, t-SNE, and ZIFA and were raw counts for others.

**Fig. 2:**
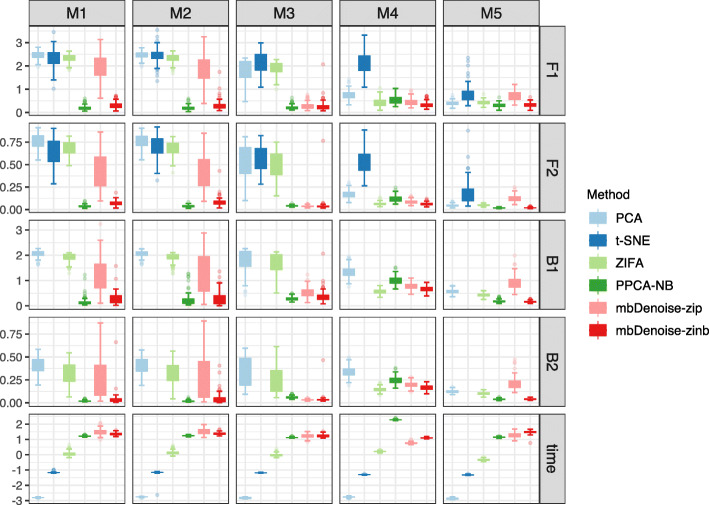
Boxplots of error measures of estimation and prediction for mbDenoise and other methods. F1 and F2 denote the orthogonal projection distance and symmetric Procrustes error for predicting ***f***_*i*_, B1 and B2 represent those for estimating ***β***_*j*_, all averaged over 100 data replications, and time is the average computation time in seconds on the log base 10 scale. Absence of results for t-SNE in B1 and B2 was due to no estimation of ***β***_*j*_ in t-SNE

The results for settings with *n*<*p* are shown in Fig. 2, and those for settings with *n*>*p* in Additional file 1: Fig. S2.1. Some observations can be made as follows. First, algorithm-based methods were outperformed by model-based methods, especially in examples M1–M4, highlighting the importance of considering data characteristics. Second, methods based on the negative binomial distribution (mbDenoise-zinb and PPCA-NB) were superior to and more robust than those based on Poisson or log normal (mbDenoise-zip and ZIFA), which makes clear the crucial role of overdispersion. As expected, model-based methods were more time-consuming than algorithm-based methods.

mbDenoise-zinb and PPCA-NB can be extended to handle the regression problem in which there are one or more covariates, denoted by mbDenoise-zinb-cov and PPCA-NB-cov. In the supplementary, we carried out additional simulations to compare them with other methods. From Additional file 1: Fig. S2.2 and S2.3 we see that the performance of mbDenoise-zinb-cov and PPCA-NB-cov were similar and among the best. To sum up, in terms of estimation and prediction, mbDenoise-zinb and mbDenoise-zinb-cov performed well.

### mbDenoise produces more reliable estimation of compositions than other methods

To measure the difference between estimated and true underlying compositions, we used Frobenius norm error, average Kullback–Leibler divergence, Shannon’s index mean squared error, and Simpson’s index mean squared error. We considered the examples M1-M3 and M6, and compared the performance of mbDenoise to that of six methods: (1) zero replacement (zr) that replaces zeros with 0.5 and then renormalizes each sample to sum one [22]. zr is simple and widely used, but ad hoc with no theoretical guarantee; (2) a version of matrix denoising known as singular value thresholding (svt) [23]; (3) Poisson-multinomial regularization (pmr) [15], which is a variant of low-rank Possion matrix recovery; (4) a Bayesian method based on Dirichlet multinomial mixtures (dmm) [24]; (5) PPCA-NB, for which compositions were constructed in the same way as they were for mbDenoise-zinb; and (6) SAVER followed by zr (SAVER_zr).

**Fig. 3:**
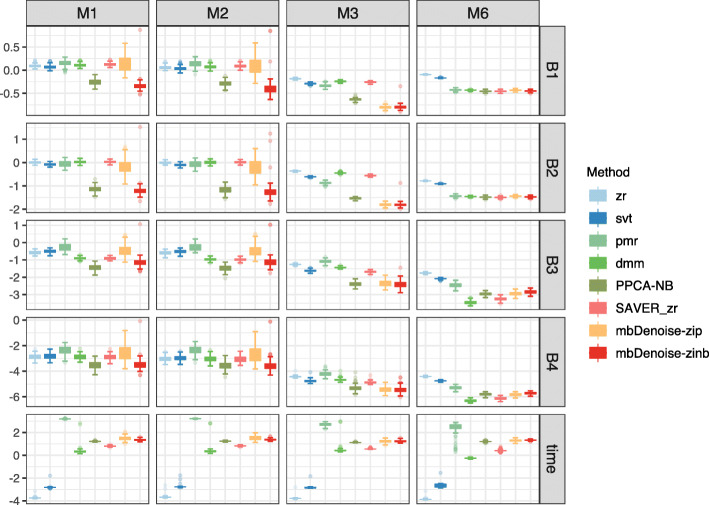
Boxplots for error measures of composition estimation for mbDenoise and other methods. C1-C4 denote the Frobenius norm error, Kullback–Leibler divergence, Shannon’s index mean squared error, and Simpson’s index mean squared error, and time stands for computation time in seconds, all averaged over 100 data replications and on the log base 10 scale

Simulation results for settings with *n*<*p* are shown in Fig. 3, and those for settings with *n*>*p* in Additional file 1: Fig. S2.4. On average, mbDenoise-zinb and PPCA-NB performed the best in estimating the compositions in examples M1-M3, and performed well compared to the best that was done in M6. As expected, pmr performed well in the multinomial example M6 without zero-inflation. Moreover, dmm and SAVER_zr tended to behave similarly with pmr, showed superior performance in example M6, but were adversely affected by zero-inflation in examples M1–M3. Though zr and svt had the poorest performance, they were computationally much cheaper.

In the supplementary, we conducted a small simulation study in which there was an environmental factor. We compared mbDenoise-zinb-cov, mbDenoise-zip-cov, and PPCA-NB-cov with zr, svt, pmr, dmm, and SAVER_zr. Additional file 1: Fig. S2.5 and S2.6 show that the performance of mbDenoise-zinb-cov was again among the best.

### mbDenoise outperforms other methods in recovering data and empowers DA analysis

Next, we compared mbDenoise with mbImpute and SAVER, in terms of how they recovered the true abundance levels, by generating simulation data from examples M1–M5. To measure the difference between the imputed/denoised matrix and the signal matrix, we used three metrics: mean squared error between the log of denoised matrix and the log of signal matrix, mean of taxon-wise Pearson correlation between the denoised matrix and the signal matrix, and Wasserstein distance between the mean community composition of denoised data and that of true abundance data.

**Fig. 4:**
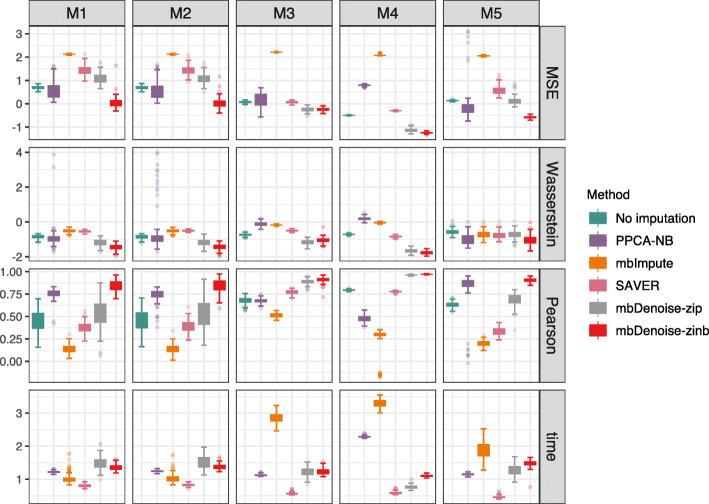
Boxplots for error measures of data recovery for mbDenoise and other methods. MSE and Wasserstein: mean squared error and Wasserstein distance between the denoised matrix and the signal matrix, averaged over 100 data replications and on the log base 10 scale. Pearson: average of Pearson correlation between the denoised and true abundance data. time: average of computation time in seconds on the log base 10 scale

Figure 4 and Additional file 1: Fig. S2.7 show the results for settings with *n*<*p* and *n*>*p*, respectively. Somewhat surprisingly, mbImpute failed badly and performed substantially worse than no imputation, suggesting that it under- or over-imputed the data. SAVER had similar behavior to mbImpute. The performance of PPCA-NB and mbDenoise-zip was mixed. PPCA-NB was outperformed by mbDenoise-zinb in examples M1–M4, and mbDenoise-zip showed inferior performance to mbDenoise-zinb in M1, M2, and M5. Overall, mbDenoise-zinb achieved the best performance, suggesting the benefit of introducing zero-inflation and overdispersion. Similar to mbDenoise-zinb, mbDenoise-zinb-cov showed superior performance in the presence of an environmental factor; see additional simulations in the supplementary.

We can view imputation/denoising as a way of normalizing the data. By removing biases introduced in sample collection, library preparation, and sequencing, the normalized data can reflect the underlying biology. In order to evaluate whether imputation/denoising has effectively removed such biases, we extended examples M1–M3 to M7–M9 in the supplementary, and examined DA testing between two groups by simply applying Welch’s *t* test to the log-transformed imputed/denoised data. We also examined the performance of existing DA analysis methods, including two negative binomial based tests for RNA-seq data, DESeq2 and edgeR, and a zero-inflated Gaussian based test for use on microbiome data, metagenomeSeq. We used the built-in normalization and default parameters. Finally, *t* test without normalization was also provided here for comparison.

**Fig. 5:**
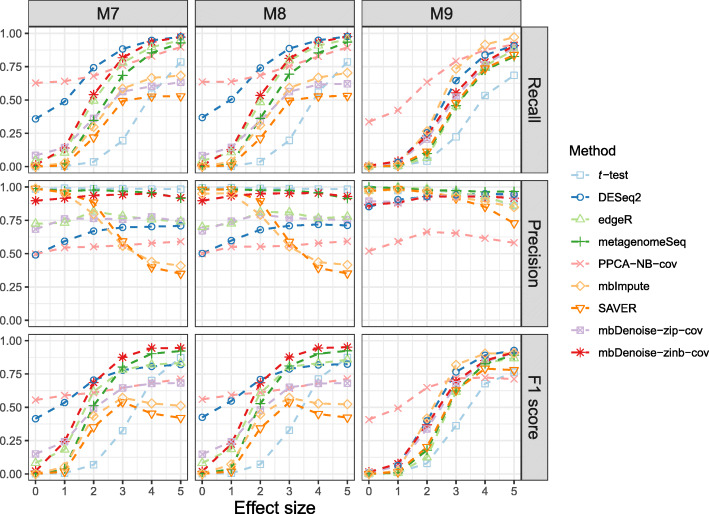
Recall, precision, and F1 score for various testing methods across different effect sizes, each averaged over 100 data replications. We used a FDR threshold of 0.05. Full details of the data generation are given in the supplementary

For settings with *n*<*p*, the precision, recall, and F1 score for various methods are shown in Fig. 5. Several points are worth noting about the results. First, without any normalization, *t* test had dramatically low recall and so was not recommended, which makes it clear that some sort of normalization was needed. Second, the recall for DESeq2 and edgeR was higher than *t*-test, but the precision was alarmingly low. It has been reported in the literature that DESeq2 and edgeR both have unexpectedly high false discovery rates for detecting differentially abundant taxa [1] and for identifying differentially expressed genes [25]. This is likely due to each method’s built-in normalization process. Studies have shown that methods developed specifically for RNA-seq data are not suitable for microbiome data [1]. Third, the recall for mbImpute and SAVER was higher than *t*-test in most cases, but the precision decreased as the effect size increased. This suggests that improper denoising/imputation could lead to false discoveries and we should proceed with caution. In particular, SAVER treats all zeros equally and removes extremely low-abundance features at the beginning, and hence it fails to distinguish technical zeros from biological zeros, leading to inaccurate abundance/expression estimates. Fourth, mbDenoise-zinb-cov maintained high recall and high precision and achieved the best F1 score. In contrast, the precision of PPCA-NB-cov was the lowest under most conditions. Failure to account for zero-inflation was the main reason for the poor performance of PPCA-NB-cov. On the other hand, the superior performance of mbDenoise-zinb-cov over the other two methods based on zero-inflated models, metagenomeSeq and mbDenoise-zip-cov, demonstrates the beneficial effect of addressing overdispersion. To summarize, normalization by mbDenoise-zinb-cov improved the performance of DA testing.

We further compared our denoising method with two popular normalization methods, cumulative-sum scaling (CSS, Paulson et al. [26]) and trimmed mean of *M* values (TMM, Robinson et al. [27]), using simulated data from examples M7–M9 with the same setup as in Fig. 5. For the sake of fairness, we applied Welch’s *t* test to the log-transformed normalized data. We also examined ANCOM [28] and the method of applying the zero-inflated negative binomial model (denoted by ZINB, Zeileis et al. [29]). Additional file 1: Fig. S2.10 shows that the proposed method mbDenoise-zinb-cov outperformed ZINB, ANCOM, CSS and TMM. Finally, Additional file 1: Table S2.1 shows that, when data were generated by SparseDOSSA 2, mbDenoise-zinb-cov performed well compared to the best that was done in each case.

### Study on stool microbiomes across geographical locations

In recent years, large-scale human microbiome projects have revealed the variability of intestinal microbial compositions in healthy individuals caused by geography, lifestyle, and other factors [30–32]. India has the second largest population in the world, whose population spread across multiple geographical locations. Different regions are typically accompanied with different dietary habits. For example, diet of Bhopal, a city of North-Central India, is mainly vegetarian or plant-based that consists of carbohydrate-rich food, while that of Kerala in Southern India is omnivorous or animal-based, consisting of protein-rich food like fish, meat, and rice.

**Fig. 6:**
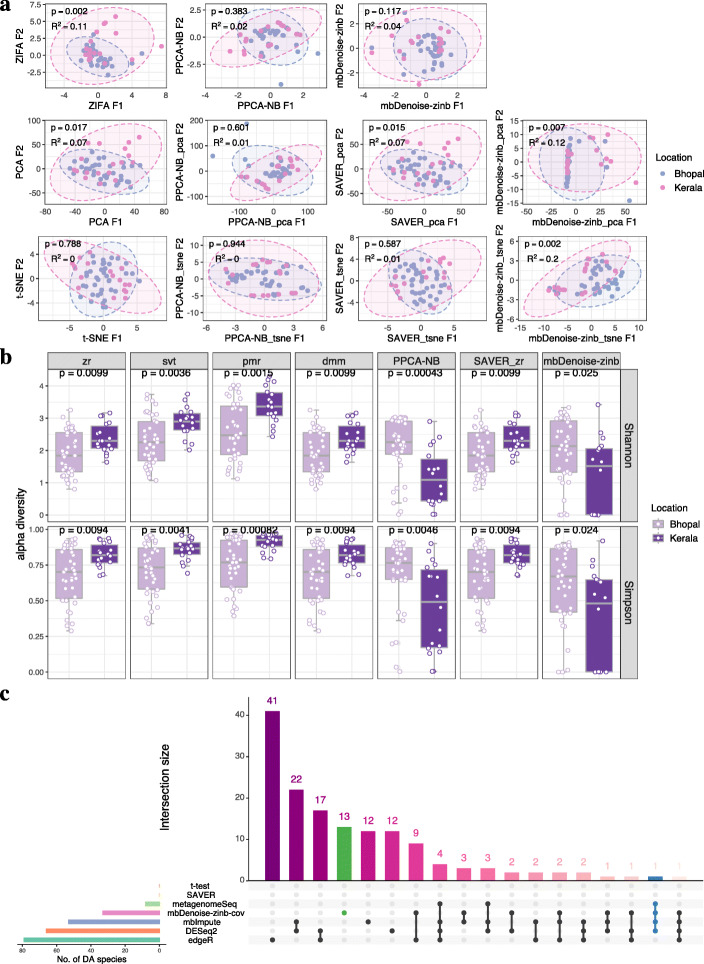
Analysis of stool microbiomes of two locations, Bhopal and Kerala, in India. **a** Data ordination through algorithm-based (PCA and t-SNE), model-based (ZIFA, PPCA-NB, mbDenoise-zinb), and denoising methods (PPCA-NB, mbDenoise-zinb and SAVER) by applying PCA and t-SNE to the denoised data. Inputs of PCA and t-SNE were log-transformed. Beta diversity was assessed using permutational multivariate analysis of variance (PERMANOVA). **b** Alpha diversity analysis. Included methods for composition estimation were zr, svt, pmr, dmm, empirical Bayes estimate by PPCA-NB and by mbDenoise-zinb, and zr using the denoised data from SAVER (SAVER_zr). Significance was calculated using the Wilcoxon test. **c** Visualization of sets of differentially abundant (DA) species between Bhopal and Kerala. Shown are sets detected by *t* test, DEseq2, edgeR, metagenomeSeq, and *t*-test applied to imputed/denoised data (mbImpute, SAVER, and mbDenoise-zinb-cov). We used a FDR threshold of 0.05

Consider the first dataset in Table 1 comprising of two locations, Bhopal and Kerala. The data, which was a subset of the microbiome survey carried out by Dhakan et al. [33], represented subjects with normal weight by body mass index (BMI, 18.5–24.9 kg/m^2^). Ordination and beta diversity analysis in Fig. 6a shows evidence of community dissimilarity between Bhopal and Kerala, which is what we expected. We also see that applying PCA and t-SNE to log-transformed denoised matrix from mbDenoise-zinb (mbDenoise-zinb_pca and mbDenoise-zinb_tsne) performed better than both intrinsic ordination in mbDenoise-zinb, and PCA and t-SNE with log-transformed count data. In other words, beta diversity analysis benefited greatly from noise reduction by mbDenoise. In contrast, the denoising by PPCA-NB and SAVER had only a negligible effect for this dataset.

**Table 1:** Summary of microbiome datasets used in empirical analysis

Dataset	Author	Publish year	Site	Level	No. of samples	No. of features	Group
Dataset 1 [33]	Dhakan et al.	2019	Stool	Species	57	235	Bhopal and Kerala
Dataset 2 [58]	Galimanas et al.	2014	Subg, supra, and tongue	Species	72	70	CP and control; site
Dataset 3 [66]	Zeller et al.	2014	Stool	Species	64	498	CRC and control
Dataset 4 [67]	Feng et al.	2015	Stool	Species	35	422	CRC and control
Dataset 5 [68]	Yu et al.	2015	Stool	Species	128	533	CRC and control
Dataset 6 [69]	Vogtmann et al.	2016	Stool	Species	56	444	CRC and control

Figure 6b shows that, for most methods for composition estimation, alpha diversity differed significantly by location, using both Shannon’s index and Simpson’s index. Interestingly enough, the empirical Bayesian estimates from mbDenoise-zinb and PPCA-NB showed the opposite result that alpha diversity of Bhopal (carbohydrate-rich diet) samples was higher than that of Kerala (protein-rich diet) samples, which seems more reasonable and is consistent with previous observations [34, 35].

In addition to the overall patterns in microbiome variation, we also assessed differential abundance using existing testing methods and methods that applied *t* test to imputed or denoised data. The number of species identified by mbDenoise-zinb-cov ranked the fourth (Fig. 6c). Note that *Prevotella copri* (blue marked), which has been acknowledged as a potential biomarker for diet [36, 37], was not identified by *t* test, SAVER, and edgeR.

**Fig. 7:**
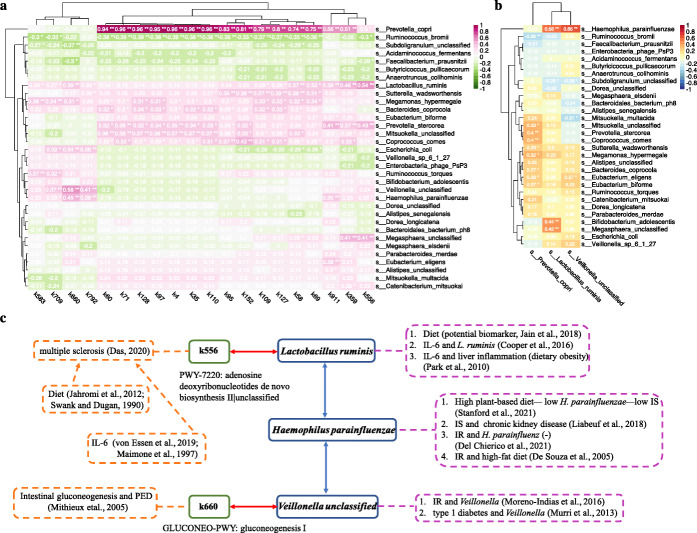
Biological function analysis of stool microbiomes of Bhopal and Kerala. **a** Heatmap of Spearman rank correlation between DA species identified by mbDenoise-zinb-cov and top twenty differential pathways found by LEfSe. k583: PWY-1269:CMP-3-deoxy-D-manno-octulosonate biosynthesis I; k709: PRPP-PWY:superpathway of histidine, purine, and pyrimidine biosynthesis; k660: GLUCONEO-PWY:gluconeogenesis I; k792: PWY-5918:superpathay of heme biosynthesis from glutamate; k60: PWY-6700:queuosine biosynthesis|g_Prevotella.s_Prevotella_copri; k71: PWY-7221:guanosine ribonucleotides de novo biosynthesis|g_Prevotella.s_Prevotella_copri; k128: PWY-2942:L-lysine biosynthesis III|g_Prevotella.s_Prevotella_copri; k97: PWY-6151:S-adenosyl-L-methionine cycle I|g_Prevotella.s_Prevotella_copri; k4: UNINTEGRATED|g_Prevotella.s_Prevotella_copri; k35: PWY-7219:adenosine ribonucleotides de novo biosynthesis|g_Prevotella.s_Prevotella_copri; k110: PWY-5097:L-lysine biosynthesis VI|g_Prevotella.s_Prevotella_copri; k95: PWY-6151:S-adenosyl-L-methionine cycle I; k152: NONMEVIPP-PWY:methylerythritol phosphate pathway I; k109: PWY-5097:L-lysine biosynthesis VI; k127: PWY-2942:L-lysine biosynthesis III; k58: PWY-6700:queuosine biosynthesis; k69: PWY-7221:guanosine ribonucleotides de novo biosynthesis; k911: PWY-5505:L-glutamate and L-glutamine biosynthesis|unclassified; k359: PWY-7229:superpathway of adenosine nucleotides de novo biosynthesis I|unclassified; k556: PWY-7220:adenosine deoxyribonucleotides de novo biosynthesis II|unclassified. **b** Heatmap of Spearman rank correlation between the three DA species most correlated with function pathways and the remaining DA species detected by mbDenoise-zinb-cov. We used FDR-adjusted *P* values. *Adjusted *P* values were between 0.01 and 0.05, **Adjusted *P* values were less than 0.01. **c** Flow chart of biological function analysis based on literature research on three DA species and two differential pathways

We extracted the corresponding function pathway data from Dhakan et al. [33], transformed the data into relative abundances using the zero replacement method, and carried out linear discriminant analysis effect size (LEfSe) to detect differential pathways between Bhopal and Kerala. The correlation heatmap between the top twenty differential pathways and the DA species identified by our method is shown in Fig. 7a. Using a cutoff of 0.5, *Prevotella copri*, *Lactobacillus ruminis*, and *Veillonella unclassified* were the three most correlated. Note that *Prevotella copri* was highly correlated with the majority of pathways. We then calculated the correlation of these three species with other DA species (Fig. 7b). We see that *Haemophilus parainfluenzae* had the highest correlation with both *Lactobacillus ruminis* and *Veillonella unclassified*.

*Lactobacillus ruminis* was identified to be a potential biomarker of diet [37]. In addition, significant enrichment of *Lactobacillus ruminis* was found in high Interleukin-6 (IL-6) producers [38]. IL-6 is a pro-inflammatory cytokine, which is associated with diabetes and obesity. Moreover, the development of liver cancer promoted by obesity depends on the production of tumor promoting cytokines IL-6 and TNF, which can cause liver inflammation and activation of oncogenic transcription factor STAT3 [39]. The pathway k556 most correlated with *Lactobacillus ruminis* was reported to be implicated in multiple sclerosis [40], which is closely related to diet [41, 42] and IL-6 [43, 44]. On the other hand, *Haemophilus parainfluenzae* was negatively associated with high plant-based diet, and was linked to elevated total indoxyl sulfate (IS) levels [45]. IS was then reported in connection with adverse clinical complications in patients with chronic kidney disease [46]. Furthermore, *Haemophilus parainfluenzae* was considered to have a negative correlation with insulin resistance (IR) [47], which can be activated by high-fat diet [48]. Conversely, there was a significant increase in the abundance of *Veillonella* at genus level (to which species *Veillonella unclassified* belongs) with IR [49] and type 1 diabetes [50]. Bizarrely, the positive correlation between *Haemophilus parainfluenzae* and *Veillonella unclassified*, and the negative and positive correlations between IR and *Haemophilus parainfluenzae* and *Veillonella*, respectively, are contradictory, which are worthy of more research. Interestingly, the pathway K660 most correlated with *Veillonella unclassified* concerns the process of intestinal gluconeogenesis, whose portal sensing is a clinical link in the diminution of food intake induced by protein-enriched diet (PED) [51]. Although there is a still debate about PED that promotes satiety, weight loss and glucose homeostasis, it may be the basis for new nutritional strategies to tackle the severe metabolic consequences of obesity and diabetes. Figure 7c presents a flow chart of biological function analysis discussed thus far.

Thus, in addition to *Prevotella copri* and *Lactobacillus ruminis*, we argued that *Haemophilus parainfluenzae* and *Veillonella unclassified* are potential biomarkers. Note that *Haemophilus parainfluenzae* and *Veillonella unclassified* were uniquely identified by our method. Other species uniquely detected by mbDenoise-zinb-cov (green marked in Fig. 6c) included *Prevotella stercorea*, *Eubacterium eligens*, and *Alistipes senegalensis*. Like *Prevotella copri*, *Prevotella stercorea* belongs to *Prevotella* genus, which is related to plant-rich diet [32, 52, 53], *Eubacterium eligens* was reported to be negatively associated with dietary fructose intake [54], and *Alistipes senegalensis* belongs to *Alistipes* genus, which is bile-tolerant and abundant in animal-based diet [31].

To obtain convincing evidence that overall patterns in microbiome variation and DA species identified between two locations were not biases introduced by mbDenoise, we extracted the subset of the data from Bhopal (39 samples and 176 species), and carried out a negative control experiment by randomly assigning a binary label (Bhopal/Kerala) to each sample and then repeating the analysis. Additional file 1: Fig. S3.1 shows that there was no significant difference in community composition, for all methods except PPCA-NB, and that our method declared far fewer DA species than DESeq2, edgeR, and mbImpute did.

### Study on oral microbiomes of chronic periodontitis

Periodontitis is an inflammatory disease that leads to the destruction of tooth-supporting tissues. The main type of periodontal disease is chronic periodontitis (CP), which is the leading cause of adult tooth loss in the world [55]. The pathogenesis of periodontitis is not only affected by genetic and epigenetic factors, but is also regulated by the formation of microbial biofilms on and around teeth [56, 57].

**Fig. 8:**
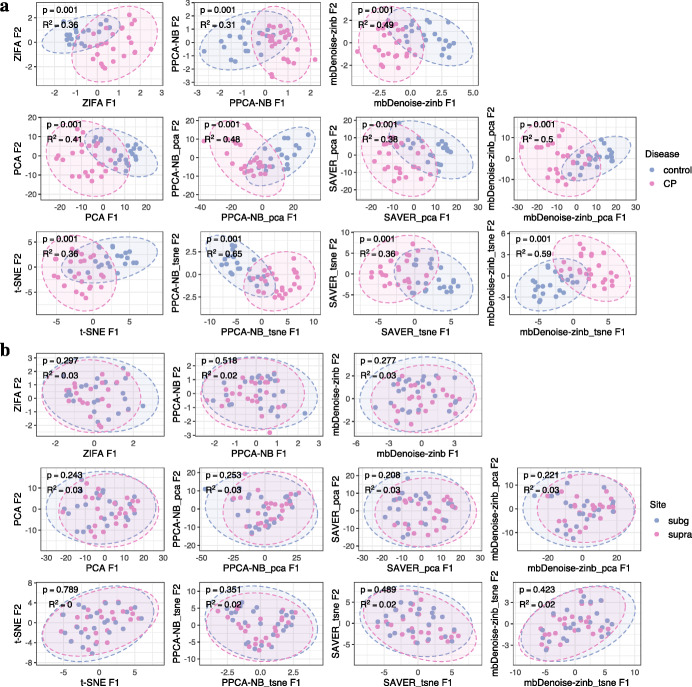
Analysis of oral microbiomes of chronic periodontitis. **a** Data ordination and beta diversity analysis between CP patients and healthy controls, with tongue samples removed. **b** Data ordination and beta diversity analysis between subg and supra sites. Included were algorithm-based (PCA and t-SNE), model-based (ZIFA, PPCA-NB, mbDenoise-zinb), and denoising methods (PPCA-NB, mbDenoise-zinb and SAVER) by applying PCA and t-SNE to the denoised data. Inputs of PCA and t-SNE were log-transformed. Beta diversity was assessed using PERMANOVA

Consider the second dataset in Table 1 from a study by Galimanas et al. [58] in which microbial samples were collected from both CP patients and healthy controls and across three oral sites, tongue, below the gingiva (subg), and above the gingiva (supra). Ordination and beta diversity analysis in Additional file 1: Fig. S3.2a reveals significant community distinctions between CP and control groups, and between gingiva and tongue, but hardly any difference between subg and supra sites. To simplify matters, we restricted attention to gingival sites. The corresponding data subset consisted of 48 samples and 70 species. Figure 8a and b show that there was again significant difference in community structure between CP and control groups, but no difference between subg and supra sites.

**Fig. 9:**
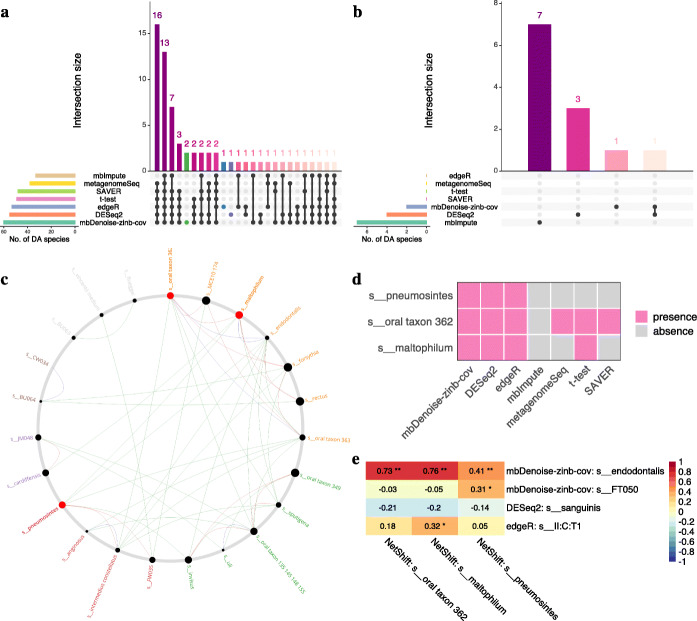
DA testing and network analysis on gingival microbiomes of chronic periodontitis. **a** Visualization of sets of DA species between CP and control groups. Shown are sets detected by *t*-test, DEseq2, edgeR, metagenomeSeq, and *t*-test applied to imputed/denoised data (mbImpute, SAVER, and mbDenoise-zinb-cov). We used a FDR threshold of 0.05. **b** Visualization of sets of DA species between subg and supra sites. **c** The most common sub-network between CP patients and healthy controls using NetShift. Drivers were red marked. **d** Spread of drivers for each DA detection method. **e** Spearman rank correlation between drivers and unique DA species. *Adjusted *P* values were between 0.01 and 0.05, **Adjusted *P* values were less than 0.01

We then turned to the question of identifying microbial taxa that explain differences between CP and control samples, which may serve as indicators of progress for CP. We compared our method with others using the same subsets as in Fig. 8a and b. The set visualization in Fig. 9a shows that mbDenoise-zinb-cov identified the largest number of species between CP and control samples. Moreover, two species, *Porphyromonas endodontalis* and *Selenomonas FT050*, were identified by mbDenoise-zinb-cov uniquely (green marked). Some studies indicate that *Porphyromonas endodontalis* is likely to be implicated in CP [59, 60]. *Selenomonas FT050* was found to have a high level in generalized aggressive periodontitis [61], suggesting that it may act as a bridge between generalized aggressive periodontitis and CP. On the other hand, Fig. 9b shows that there were few or no DA species between subg and supra sites for most methods.

DA species are not only reflected in community composition and diversity, but also in the variation of microbe-related network topology. In order to evaluate the reliability of DA species detected by our method, we applied NetShift [62] to quantify changes in microbial association network between CP and control groups. We first calculated the correlation matrix of species separately for CP patients and healthy controls. Then the edge matrices were constructed and used as inputs into NetShift. Figure 9c shows the most common sub-network. We see that there were three microbe drivers (red marked), *pneumosintes*, *oral taxon 362*, and *maltophilum*. Moreover, these drivers were also DA species identified by mbDenoise-zinb-cov, DESeq2, and edgeR, were only partly detected by metagenomeSeq, *t* test, and SAVER, and were completely missed by mbImpute (Fig. 9d). We then calculated the Spearman rank correlation between these drivers and unique DA species identified by mbDenoise-zinb-cov, DESeq2, and edgeR. Using a cutoff of 0.5, Fig. 9e shows that only *Porphyromonas endodontalis* (uniquely identified by mbDenois-zinb-cov) was highly correlated with two drivers *maltophilum* and *oral taxon 362*.

We also extracted the data subset, comprising of tongue samples from CP patients and healthy controls, and carried out alpha diversity analysis. Additional file 1: Fig. S3.2b suggests that only our proposed method performed well, being consistent with previous studies that patients with chronic periodontitis were associated with significantly higher alpha diversity than those for healthy subjects [63].

### Study on stool microbiomes of colorectal cancer

Colorectal cancer (CRC) is the third most commonly diagnosed human malignant tumor and the fourth highest cause of cancer-related death worldwide [64]. There is increasing evidence that intestinal microbiota dysbiosis plays a pivotal role in the development of colorectal cancer [65].

**Fig. 10:**
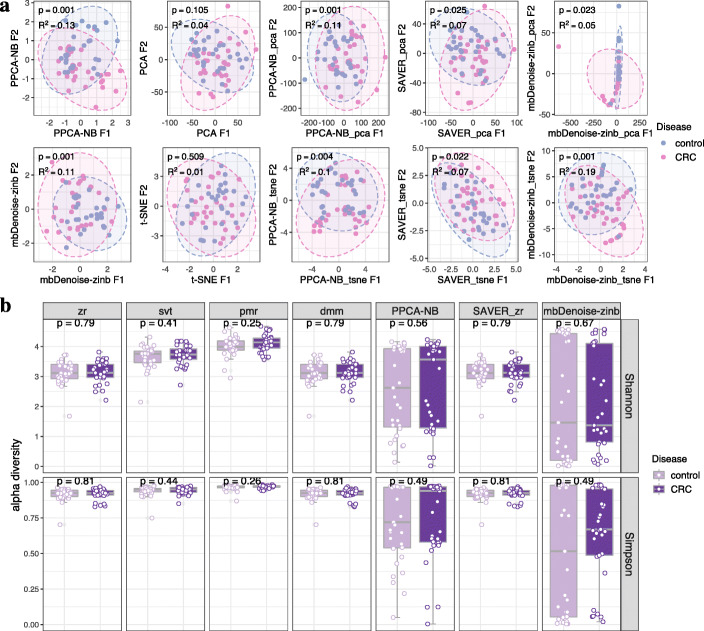
Analysis of stool microbiomes of colorectal cancer. **a** Data ordination through algorithm-based (PCA and t-SNE), model-based (ZIFA, PPCA-NB, mbDenoise-zinb), and denoising methods (PPCA-NB, mbDenoise-zinb and SAVER) by applying PCA and t-SNE to the denoised data on the third dataset in Table 1. Inputs of PCA and t-SNE were log-transformed, and the empty was due to an exception in ZIFA. Beta diversity was assessed using PERMANOVA. **b** Alpha diversity analysis on the third dataset in Table 1. Included methods for composition estimation were zr, svt, pmr, dmm, empirical Bayes estimate by PPCA-NB and by mbDenoise-zinb, and zr using the denoised data from SAVER (SAVER_zr). Significance was calculated using the Wilcoxon test

Consider the third to sixth datasets in Table 1, each comprising of CRC and healthy control samples. These datasets were subsets of microbiome surveys carried out by Zeller et al. [66], Feng et al. [67], Yu et al. [68], and Vogtmann et al. [69], representing subjects with normal weight by BMI (18.5–24.9 kg/m^2^). Ordination and beta diversity analysis in Fig. 10a and Additional file 1: Fig. S3.3 show that for the third to fifth datasets CRC patients could be roughly distinguished from healthy controls. This is especially the case for model-based methods. On the other hand, we see from Fig. 10b and Additional file 1: Fig. S3.4 that there was no discernible difference in alpha diversity between the two groups, with the exception of mbDenoise-zinb in the fifth dataset having the largest sample size. In this case, only mbDenoise-zinb arrived at a conclusion that CRC patients had significantly decreased alpha diversity compared with healthy subjects, which is consistent with previous findings [70].

**Fig. 11:**
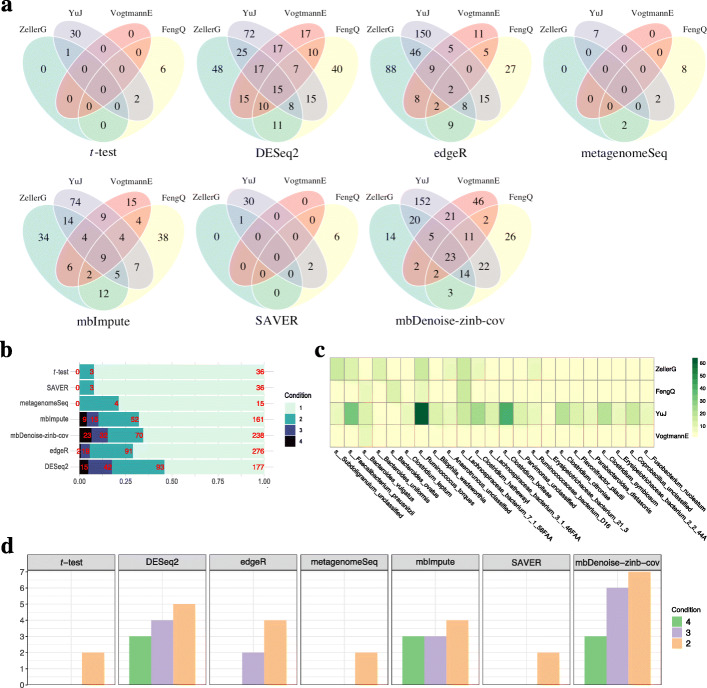
DA analysis of stool microbiomes of colorectal cancer. **a** Venn diagrams of DA species across four CRC datasets. Shown are sets detected by *t*-test, DEseq2, edgeR, metagenomeSeq, and *t*-test applied to imputed/denoised data (mbImpute, SAVER, and mbDenoise-zinb-cov). We used a FDR threshold of 0.05. **b** Percent stacked barplot of CRC-enriched species identified across four datasets by each method. Condition 1-4 represent species identified in one, two, three, and four datasets. Numbers marked red correspond to the counts found in each condition. **c** Heatmap of − log10(FDR-corrected *P* values) of the 23 common species identified by mbDenoise-zinb-cov across four datasets. **d** The frequencies of seven CRC biomarkers discovered across four datasets in each method. Condition 4-2 represent biomarkers identified in four, three, and two datasets

Different from DA analysis on the first and second datasets, here we aimed to demonstrate the reproducibility of our method by using four CRC datasets. The Venn diagrams in Fig. 11a show that mbDenoise-zinb-cov identified the largest number of species. Furthermore, it had the best reproducibility in the sense that 23 out of 363 species were found in all four datasets (Fig. 11b). DESeq2 did the second best, but the results from a negative control study (Additional file 1: Fig. S3.5) indicate that it was overly liberal. We note that the 23 common species identified by mbDenoise-zinb-cov (Fig. 11c) mainly belong to five families (*Clostridiaceae*, *Ruminococcaceae*, *Bacteroidaceae*, *Lachnospiraceae*, and *Erysipelotrichaceae*), which have been reported to be associated with CRC [71–74]. More importantly, seven previously reported CRC biomarkers, including *Fusobacterium nucleatum*, *Faecalibacterium prausnitzii*, *Bacteroides fragilis*, *Peptostreptococcus stomatis*, *Parvimonas micra*, *Solobacterium moorei*, and *Clostridium symbiosum* [75–78], were discovered most frequently by mbDenoise-zinb-cov (Fig. 11d).

Several other DA species in Fig. 11c are also worthy of mention. These include *Bacteroides vulgatus*, *Ruminococcus torques*, *Clostridium hathewayi*, *Clostridium bolteae*, and *Lachnospiraceae* bacterium 7_1_58FAA. *Bacteroides vulgatus* is enriched in the gut microbiota of healthy people compared with CRC patients [79], and evidence suggests that it is associated with Crohn’s disease (CD) [80]. *Ruminococcus torques* was reported to increase disproportionately in patients with CD and ulcerative colities [81], both of which increase the risk of developing CRC [82]. It is also correlated with high red meat intakes that contribute to an increased risk of CRC [83]. *Clostridium hathewayi*, combined with *Fusobacterium nucleatum* and two other bacteria, improve diagnostic performance of *Fusobacterium nucleatum* alone [84, 85]. *Clostridium bolteae* is identified to drive lipopolysaccharide biosynthesis in the gut of CRC patients [86]. *Lachnospiraceae* bacterium 7_1_58FAA has a clear connection with L-glutamate degradation V. Note that L-glutamate dehydrogenase deficiency leads to D-2-hydroxyglutarate dehydrogenase deficiency [87], and a CRC specific pathway through D-2-hydroxyglutarate can drive epithelial-mesenchymal transition and induce CRC progression [88].

## Discussion

The work in this paper connects to the recent interest in modeling microbiome count data. Specifically, our proposed ZIPPCA-NB model and those of Xu et al. [89] and Sohn and Li [90] all belong to the general class of generalized latent variable models. However, the latter two methods treat the ***f***_*i*_’s as unknown parameters to be estimated rather than random variables. Another difference concerns the underlying count distribution. It is well-known that the negative binomial distribution in ZIPPCA-NB is preferable to Poisson [89] or quasi-Poisson [90] in terms of addressing the overdispersion of microbiome count data. Nevertheless, there is a price to be paid for the increased flexibility of ZIPPCA-NB, which requires computationally intensive numerical optimization techniques. Recent work by Liu et al. [91] also uses the negative binomial distribution as the underlying count distribution, and applies variational approximation (VA) for approximate estimation and inference. However, there are important differences between mbDenoise and their method, MZINBVA. First, mbDenoise is a general statistical method for describing and simulating microbial community profiles in a cross-sectional study, whereas MZINBVA focuses on differential abundance testing in longitudinal/multi-level studies. Second, mbDenoise fits the data at the overall community level with rich dependencies among taxa and independence among samples, whereas MZINBVA fits the data at the individual taxon level accounting for structured correlations between samples but ignoring correlations between taxa.

Rather than using sampling, the main idea behind VA is to use optimization. However, inference using VA is challenging. Nevertheless, some progress has been made in the literature. Assume that the dimension of the units of analysis tends to infinity, but that the other dimension of features is fixed, Westling and McCormick [92] build a connection between VA and profile *M*-estimation, and provide a sandwich covariance formula for the VA estimate; see also Liu et al. [91]. However, due to the complicated nature of the problem, developing a general theory in high dimensions would be a substantial undertaking that the result would effectively be a separate paper. Work along this line is in progress. An alternative is to use the Laplace approximation or penalized quasi-likelihood. However, compared with VA, the Laplace approximation is known to suffer from convergence problems [20].

As pointed out by one referee, a common approach to address data redundancy is to take into account phylogenetic tree information. For example, the Unifrac method incorporates tree information and can be used in ordination and distance-based testing approaches such as PERMANOVA [93]. There are two reasons why we do not consider the phylogenetic relationships among microbes. First, incorporating the tree structure into the ZIPPCA model underlying mbDenoise will inevitably make the current modeling and fitting too complicated. Second, in practice the phylogeny is inferred from molecular sequences, and so it is necessary to quantify uncertainty in phylogenetic inference and its impact on downstream analyses. Nevertheless, it is interesting yet challenging to describe and simulate microbial community profiles while taking into account the phylogenetic tree information.

Care must be taken during denoising because one can never rule out the possibility that signal may be lost from the data. This is likely a consequence of the linearity nature of the ZIPPCA model, that is, $ \log \mu _{ij}=\alpha _{i0}+\beta _{0j}+\boldsymbol {f}_{i}^{\top }\boldsymbol {\beta }_{j}.$ In practice, the assumption of linearity is questionable and nonlinear functions of ***f***_*i*_ such as neural networks might do a better job.

mbDenoise could facilitate other forms of downstream analysis not considered in this paper. One such task is the inference of microbial correlation networks [94, 95]. Unfortunately, technical noise in microbiome data makes it challenging to quantify dependencies or interactions between microbes [96, 97]. Denoising has the potential to enhance the discovery of these interactions. Some progress on this problem has been made in the field of single-cell transcriptomics. van Dijk et al. [98] proposed Markov affinity-based graph imputation of cells (MAGIC) to recover gene expression values while correcting for dropout and other sources of noise, and demonstrated that MAGIC was effective at inferring gene-gene interactions. However, care must be taken when carrying out correlation analysis, as with other high-level analyses, because over-denoising may obscure important relationships and introduce spurious correlations between genes [12]. To our knowledge, the performance of computational and statistical methods for inferring microbial ecological networks from denoised data has not been evaluated using either simulated or real datasets, which is a necessary step on the road to understanding the impact of denoising and represents an important direction for future research.

## Conclusion

A fundamental challenge in the analysis of microbial abundance data is technical noise. mbDenoise was proposed specifically for microbiome data to decouple biological variation from technical noise. mbDenoise is based on a zero-inflated negative binomial probabilistic PCA (ZIPPCA-NB) model that distinguishes between biological and technical zeros, and accounts for unequal library size and overdispersion of data. mbDenoise learns the parameters of ZIPPCA-NB using a highly efficient variational approximation algorithm. The low rank latent representation in the ZIPPCA-NB model, which makes use of a mild assumption of data redundancy, enables the learning process to aggregate information across samples and taxa. mbDenoise adopts an empirical Bayes approach to recover true abundance levels.

We extensively evaluated the performance of mbDenoise using simulated experiments and empirical datasets. We demonstrated that mbDenoise achieves high accuracy in estimating model parameters and predicting latent variables as well as in estimating underlying microbial compositions, and that both zero-inflation and overdispersion are essential components for its superior performance. In most cases, mbDenoise compared favorably to state-of-the-art methods in recovering true abundance levels and improving high-level analyses including unconstrained ordination, diversity estimation, and differential abundance analysis. We thus expect mbDenoise to be a nice contribution to the statistical toolbox for analyzing and interpreting microbiome data.

## Methods

### Noise model

mbDenoise is a denoising method for microbiome data based on a ZIPPCA model that addresses overdispersion and zero-inflation. First, the Gaussian distribution in probabilistic PCA for continuous variables is extended to the negative binomial distribution for describing overdispersed sequence counts. Second, a Bernoulli distribution is used to characterize excess zeros as either biological zeros (true absence) or technical zeros (undetected presence). More formally, this two-part noise model can be expressed as

$$\begin{aligned} \text{latent space} \ \ & z_{ij} \stackrel{ind}{\sim}\ Bern(\eta_{ij}),\\ &f_{i1},\ldots,f_{ik} \stackrel{ind}{\sim}\ N(0,1),\\ \text{parameter space} \ \ & \eta_{ij} = \frac{\exp (c_{i}+\tau_{j})}{1+\exp (c_{i}+\tau_{j})},\\ & \log \mu_{ij}=\alpha_{i0}+\beta_{0j}+\boldsymbol{f}_{i}^{\top}\boldsymbol{\beta}_{j},\\ \text{observation space} \ \ & x_{ij}\mid \mu_{ij},z_{ij} \stackrel{ind}{\sim} \left\{ \begin{array}{ll} 0 & \text{if} \ \ z_{ij}=1, \\ NB(\mu_{ij},\phi_{j}) & \text{if} \ \ z_{ij}=0, \end{array} \right.  \end{aligned} $$

where *ind* means independently distributed, and *Bern*, *N*, and *NB* denote the Bernoulli, normal, and negative binomial distributions, respectively. The *z*_*ij*_ are latent indicators for excess zeros, and *η*_*ij*_, the probabilities of zero inflation, are specified by a linear logit link, with sample-specific parameters *c*_*i*_ and taxon-specific parameters *τ*_*j*_, where *i*=1,…,*n* and *j*=1,…,*p*. *α*_*i*0_ and *β*_0*j*_ are similarly defined in the *NB* part with a log link, and *ϕ*_*j*_ are taxon-specific overdispersion parameters. When *ϕ*_*j*_→0, *NB* reduces to the Poisson distribution. The latent variables ***f***_*i*_=(*f*_*i*1_,…,*f*_*ik*_)^⊤^ representing the coordinates of observed data ***x***_*i*_=(*x*_*i*1_,…,*x*_*ip*_)^⊤^ in a *k*-dimensional latent space, *k*≪*p*, and the factor loadings ***β***_*j*_ jointly capture the correlations among microbes [99].

Note that, in the above, sample-specific parameters *α*_*i*0_ are introduced to handle the uneven library size across samples, and the low-rank representation $\boldsymbol {f}_{i}^{\top }\boldsymbol {\beta }_{j}$ takes advantage of the redundancy in microbiome data.

### Variational approximation for ZIPPCA

Let ***Θ***={*c*_*i*_,*τ*_*j*_,*α*_*i*0_,*β*_0*j*_,***β***_*j*_,*ϕ*_*j*_} denote the set of parameters governing the ZIPPCA model. Finding the maximum likelihood estimate is difficult, because the integrals involved in the data likelihood do not have closed form expressions. A general technique in the latent variable modeling literature is the Monte Carlo expectation maximization algorithm. However, Monte Carlo methods are computationally intensive and have mainly been used for small-scale problems. Here, we adopt a highly efficient deterministic approximation approach, known as variational approximation (VA) [100]. The main idea of VA is to specify a family of distributions and then find a member of the family that is close to the true posterior distribution of latent variables. Specifically, consider a variational family of distributions *q*(***f***_*i*_,***z***_*i*_) for the latent variables (***f***_*i*_,***z***_*i*_). Using Jensen’s inequality, the data log-likelihood satisfies

$$\begin{aligned} \sum_{i=1}^{n}\log p(\boldsymbol{x}_{i}) =&\sum_{i=1}^{n}\log \left[E_{q}\left\{\frac{p(\boldsymbol{x}_{i},\boldsymbol{f}_{i},\boldsymbol{z}_{i})}{q(\boldsymbol{f}_{i},\boldsymbol{z}_{i})} \right\} \right] \\ \notag \geq& E_{q}\sum_{i=1}^{n}\{\log p(\boldsymbol{x}_{i},\boldsymbol{f}_{i},\boldsymbol{z}_{i})\}-\sum_{i=1}^{n}E_{q}\{\log q(\boldsymbol{f}_{i},\boldsymbol{z}_{i})\}. \notag \end{aligned} $$

The right-hand size above is called an evidence lower bound (ELBO). It is easy to see that maximizing the ELBO with respect to *q*(***f***_*i*_,***z***_*i*_) is equivalent to minimizing the Kullback–Leibler divergence between *q*(***f***_*i*_,***z***_*i*_) and the true posterior *p*(***f***_*i*_,***z***_*i*_∣***x***_*i*_). The VA algorithm involves alternately computing the lower bound for the current parameter values and then maximizing this bound to obtain the new parameter values.

In this paper, we focus on the mean field variational family, where the latent variables are mutually independent [101]. Specifically, we assume *q*(*z*_*ij*_)∼*indBern*(*π*_*ij*_),*q*(***f***_*i*_)∼*indN*(***m***_*i*_,***Σ***_*i*_). Write ***π***_*i*_=(*π*_*i*1_,…,*π*_*ip*_)^⊤^ and ***Δ***={***m***_*i*_,***Σ***_*i*_,***π***_*i*_}. We call ***Δ*** the variational parameters. Denote by $\hat {\boldsymbol {\Theta }} = \{\hat {c}_{i}, \hat {\tau }_{j}, \hat {\alpha }_{i0}, \hat {\beta }_{0j}, \hat {\boldsymbol {\beta }}_{j}, \hat {\phi }_{j}\}$ and $\hat {\boldsymbol {\Delta }} = \{\hat {\boldsymbol {m}}_{i}, \hat {\boldsymbol {\Sigma }}_{i}, \hat {\boldsymbol {\pi }}_{i}\}$ the VA estimates of model and variational parameters, respectively. Details on the numerical optimization procedure can be found in the supplementary.

### Denoising

Biases or artifacts in microbiome data exist due to technical reasons, and can make downstream analyses invalid if unaddressed. Loosely speaking, denosing is a way of normalizing the data to remove technical noise, so that the denoised data are on a comparable scale. Two commonly used methods for microbiome data normalization are rarefying and scaling. However, rarefying only addresses unequal library size, and scaling is adversely affected by the large number of zeros. Furthermore, they are incapable of distinguishing between mean and dispersion effects and can cause undesirable or even erroneous results [102].

The proposed ZIPPCA-NB framework takes into account varying library sizes across samples, data sparsity and overdispersion, and data redundancy, and so leads naturally to a model-based denoising strategy. As shown in Fig. 1, the observed data *x*_*ij*_ are equal in distribution to (1−*z*_*ij*_)*NB*(*μ*_*ij*_,*ϕ*_*j*_), and the latent signal matrix that represents the underlying biological variation is defined by

$$x^{*}_{ij}=(1-z_{ij})\exp \left(\beta_{0j}+\boldsymbol{f}_{i}^{\top}\boldsymbol{\beta}_{j}\right). $$

This definition makes use of the low-rank assumption, removes sampling, sample-specific, and overdispersion effects, and distinguishes between technical and biological zeros.

mbDenoise uses the posterior mean of the latent signal matrix to recover the true abundance levels. A simple calculation shows that

$$\hat{x}^{*}_{ij}=(1-\hat{\pi}_{ij})\exp \left(\hat{\beta}_{0j}+\hat{\boldsymbol{m}}_{i}^{\top} \hat{\boldsymbol{\beta}}_{j}+\frac{1}{2}\hat{\boldsymbol{\beta}}_{j}^{\top} \hat{\boldsymbol{\Sigma}}_{i}\hat{\boldsymbol{\beta}}_{j}\right). $$

This approach is known as empirical Bayes in the literature. Note that the sample-specific effects are removed in the denoised data to eliminate the bias caused by library size.

### Dimension reduction and ordination analysis

Ordination techniques are often applied to normalized data to visually inspect whether sample groupings reflect any biological patterns in an unsupervised manner. These methods attempt to represent the main structures in multivariate community data with a reduced set of usually two or three factors.

Data ordination tends to follow one of two methodological approaches. Methods in the first group are largely algorithm-based, including PCA and t-SNE. A second and more recent approach specifies a joint model for multivariate abundance data [90]. In particular, there has been considerable interest in latent variable models, because it is natural to interpret latent variables as the factors in an ordination [21, 99].

Within the ZIPPCA framework, we can use the posterior mean or mode of ***f***_*i*_ as the ordination score. One disadvantage of this intrinsic method is that, unlike in linear PCA, solutions are not constructed incrementally [103]. Alternatively, we can adopt a general approach in which we first denoise the abundance data, and then apply an algorithm-based method to the denoised data to compute ordination axes (denoted by mbDenoise-zinb_pca and mbDenoise-zinb_tsne). We prefer the second strategy, because it not only retains the versatility of algorithm-based methods, but also accounts for the characteristics of microbiome data. As the name suggests, mbDenoise-zinb_pca and mbDenoise-zinb_tsne are proposed for linear and nonlinear dimension reduction, respectively. In real problems, the truth is unknown and patterns are often not linear, and hence we recommend mbDenoise-zinb_tsne.

### Composition estimation

Microbiome data should be considered as compositions. A common approach to extract microbial compositions from raw data is to divide every count in a sample by the total number of counts for that sample. This approach gives relative abundances that sum to one, but is problematic in the presence of many zeros, because zeros remain unchanged, which can have an undesirable effect on downstream analyses such as diversity estimation.

Methods based on a multinomial probability model have been proposed to address this issue [24]. They describe each community by a vector of taxa probabilities. However, under the ZIPPCA framework, it is not clear what we should use as a definition of composition. To this end, we note that there is a useful relationship between the Poisson and multinomial distributions [104], namely, if *x*_*j*_∼*indPoi*(*μ*_*j*_), then the conditional distribution of ***x***=(*x*_1_,…,*x*_*p*_)^⊤^ given $x_{+}=\sum _{j=1}^{p}x_{j}$ is multinomial, ***x***∣*x*_+_∼*Mult*(*x*_+_,***μ***/*μ*_+_), where ***μ***=(*μ*_1_,…,*μ*_*p*_)^⊤^ and $\mu _{+} = \sum _{j=1}^{p}\mu _{j}$. We therefore define the underlying compositions for ZIPPCA-Poi and ZIPPCA-NB as

$$\rho_{ij} = \frac{\mu_{ij}}{\sum_{k=1}^{p}\mu_{ik}}=\frac{\exp \left(\beta_{0j}+\boldsymbol{f}_{i}^{\top}\boldsymbol{\beta}_{j}\right)}{\sum_{k=1}^{p}\exp \left(\beta_{0k}+\boldsymbol{f}_{i}^{\top}\boldsymbol{\beta}_{k}\right)}. $$

For ZIPPCA-Poi, we use an empirical Bayes approach to estimate the compositions

$$\hat{\rho}^{poi}_{ij} = \frac{\exp \left(\hat{\beta}_{0j} + \hat{\boldsymbol{m}}_{i}^{\top}\hat{\boldsymbol{\beta}}_{j}+\hat{\boldsymbol{\beta}}_{j}^{\top}\hat{\boldsymbol{\Sigma}}_{i}\hat{\boldsymbol{\beta}}_{j}/2\right)}{\sum_{k=1}^{p}\exp \left(\hat{\beta}_{0k}+\hat{\boldsymbol{m}}_{i}^{\top}\hat{\boldsymbol{\beta}}_{k}+\hat{\boldsymbol{\beta}}_{k}^{\top}\hat{\boldsymbol{\Sigma}}_{i}\hat{\boldsymbol{\beta}}_{k}/2\right)}. $$

For ZIPPCA-NB, this estimate does not account for overdispersion. The problem can be resolved by first noting that the negative binomial distribution can be written as a mixture of gamma and Poisson distributions: if *x*_0*ij*_∣*w*_*ij*_∼*Poi*(*w*_*ij*_) and *w*_*ij*_∼*Gamma*(*ϕ*_*j*_,*ϕ*_*j*_/*μ*_*ij*_), then *x*_0*ij*_∣*μ*_*ij*_,*ϕ*_*j*_∼*NB*(*μ*_*ij*_,*ϕ*_*j*_). Furthermore, the optimal VA distribution for *w*_*ij*_ is a gamma distribution with shape *x*_0*ij*_+*ϕ*_*j*_ and rate $1+\phi _{j}\exp \left (-\beta _{0j}- \boldsymbol {m}_{i}^{\top }\boldsymbol {\beta }_{j}+\boldsymbol {\beta }_{j}^{\top }\boldsymbol {\Sigma }_{i}\boldsymbol {\beta }_{j}/2\right)$ [20]. The modified estimate has the form

$$\hat{\rho}_{ij}^{nb} = \frac{\left\{\exp \left(\hat{\beta}_{0j} + \hat{\boldsymbol{m}}_{i}^{\top}\hat{\boldsymbol{\beta}}_{j}+\hat{\boldsymbol{\beta}}_{j}^{\top}\hat{\boldsymbol{\Sigma}}_{i}\hat{\boldsymbol{\beta}}_{j}/2\right)+\hat{\phi}_{j}\right\}/\nu_{ij}}{\sum_{k=1}^{p}\left\{\exp \left(\hat{\beta}_{0k}+\hat{\boldsymbol{m}}_{i}^{\top}\hat{\boldsymbol{\beta}}_{k}+\hat{\boldsymbol{\beta}}_{k}^{\top}\hat{\boldsymbol{\Sigma}}_{i}\hat{\boldsymbol{\beta}}_{k}/2\right)+\hat{\phi}_{k}\right\}/\nu_{ik}}, $$

where $\nu _{ij}=1+\hat {\phi }_{j}\exp \left (-\hat {\beta }_{0j} - \hat {\boldsymbol {m}}_{i}^{\top }\hat {\boldsymbol {\beta }}_{j}+\hat {\boldsymbol {\beta }}_{j}^{\top }\hat {\boldsymbol {\Sigma }}_{i}\hat {\boldsymbol {\beta }}_{j}/2\right).$

### DA analysis

For DA testing between two groups, a naive method is to model the abundance data separately for each group, combine the denoised data, and then apply a test. However, this approach does not account for the fact that samples from different conditions may have much in common, and these similarities can be used to learn from the experience of others. Also, when the groups are unbalanced, such a strategy is likely to perform poorly. To address this, we take advantage of the regression-type formulation of the ZIPPCA framework and treat the group indicator as a covariate. The corresponding log link has the form

$$\log \mu_{ij}=\alpha_{i0}+\beta_{0j}+\gamma_{j} v_{i}+\boldsymbol{f}_{i}^{\top}\boldsymbol{\beta}_{j}, $$

where *v*_*i*_ is the covariate (e.g., healthy versus diseased), and *γ*_*j*_ is the coefficient. We use the covariate-adjusted model for fitting and denosing data, log-transform the denoised data, and then apply Welch’s *t* test to determine which specific taxa are significantly differentially abundant between two groups. Extensions to multiple groups and more than one covariate is straightforward.

### Evaluation metrics

We provide below details of various metrics or indices for assessing the performance of mbDenoise in the simulation.

**Estimation and prediction indices** Suppose **M** and $\hat {\mathbf {M}}$ are the true and estimated/predicted matrices, respectively. We use two criteria for measuring the distance between **M** and $\hat {\mathbf {M}}$.

(A1) Symmetric Procrustes error. First, center the columns of **M** by their means and rescale the centered matrix to have unit Frobenius norm. Denote the transformed matrix by **M**_*t*_. Similarly, we obtain $\hat {\mathbf {M}}_{t}$. Second, compute the singular value decomposition $\mathbf {M}_{t}^{\top }\hat {\mathbf {M}}_{t}= \mathbf {U}\mathbf {D}\mathbf {V}^{\top }$, and then construct a rotated version of $\hat {\mathbf {M}}_{t}$ as

$$\hat{\mathbf{M}}_{rot}=\frac{ trace(\mathbf{D})}{\|\hat{\mathbf{M}}_{t}\|^{2}_{F}}\hat{\mathbf{M}}_{t}\mathbf{V}\mathbf{U}^{\top}. $$

Finally, calculate the squared Frobenius matrix norm $\|\mathbf {M}_{t}-\hat {\mathbf {M}}_{rot}\|_{F}^{2}$;

(A2) Orthogonal projection distance. Let **P**_**M**_=**M**(**M**^⊤^**M**)^−1^**M**^⊤^ and $\hat {\mathbf {P}}_{\hat {\mathbf {M}}}= \hat {\mathbf {M}} (\hat {\mathbf {M}}^{\top } \hat {\mathbf {M}})^{-1} \hat {\mathbf {M}}^{\top }$ be orthogonal projections onto the column spaces of **M** and $\hat {\mathbf {M}}$, respectively. Calculate the squared Frobenius matrix norm $\|\mathbf {P}_{\mathbf {M}}-\hat {\mathbf {P}}_{\hat {\mathbf {M}}}\|_{F}^{2}$.

**Composition estimation indices** Different measures of the closeness between the true compositions *ρ*_*ij*_ and the estimated compositions $\hat {\rho }_{ij}$ include the following

(B1) Frobenius norm error: $ \sqrt {\sum _{i=1}^{n}\sum _{j=1}^{p}(\hat {\rho }_{ij}-\rho _{ij})^{2}};$

(B2) average Kullback–Leibler divergence: $(1/n)\sum _{i=1}^{n}\sum _{j=1}^{p}\rho _{ij}\log \left (\rho _{ij}/\hat {\rho }_{ij}\right);$

(B3) Shannon’s index mean squared error: $(1/n)\sum _{i=1}^{n}\left \{\sum _{j=1}^{p}\hat {\rho }_{ij}\log (\hat {\rho }_{ij})-\sum _{j=1}^{p} \rho _{ij}\log (\rho _{ij})\right \}^{2};$

(B4) Simpson’s index mean squared error: $(1/n)\sum _{i=1}^{n}\left (\sum _{j=1}^{p}\rho _{ij}^{2}-\sum _{j=1}^{p}\hat {\rho }_{ij}^{2}\right)^{2}.$

**Data recovery indices** To assess the agreement between the denoised matrix $(\hat {x}^{*}_{ij})$ and the signal matrix $(x^{*}_{ij})$, we calculate three criteria:

(C1) mean squared error between the log of denoised matrix and the log of signal matrix

$$\frac{1}{np}\sum_{i=1}^{n}\sum_{j=1}^{p}\left\{\log_{2}(x^{*}_{ij}+1)-\log_{2} (\hat{x}^{*}_{ij}+1)\right\}^{2}; $$

(C2) mean of taxon-wise Pearson correlation between the denoised and signal matrices

$$\frac{1}{p}\sum_{j=1}^{p}\frac{\hat{\text{cov}}\left(\boldsymbol{x}^{*}_{j},\hat{\boldsymbol{x}}^{*}_{j}\right)}{\hat\sigma\left(\boldsymbol{x}^{*}_{j}\right)\hat\sigma\left(\hat{\boldsymbol{x}}^{*}_{j}\right)}, $$

where $\hat {\text {cov}}$ denotes the sample covariance, and $\hat \sigma $ means the sample standard deviation;

(C3) Wasserstein distance between the mean community composition of denoised data and that of true abundance data

$$\frac{1}{p}\sum_{j=1}^{p}\left|r^{*}_{(j)}-\hat{r}^{*}_{(j)}\right|, $$

where $r^{*}_{j}=\sum _{i=1}^{n}x^{*}_{ij}/\left \{n\hat {\sigma }\left (\boldsymbol {x}^{*}_{j}\right)\right \}$ and $\hat {r}^{*}_{j}=\sum _{i=1}^{n}\hat {x}^{*}_{ij}/\left \{n\hat {\sigma }\left (\hat {\boldsymbol {x}}^{*}_{j}\right)\right \}$, and $r^{*}_{(j)}$ and $\hat {r}^{*}_{(j)}$ denote the order statistics of $\left \{r^{*}_{1},\ldots,r^{*}_{p}\right \}$ and $\left \{\hat {r}^{*}_{1},\ldots,\hat {r}^{*}_{p}\right \}$, respectively.

### Existing tools or software

We describe below some of the tools or software used in the study.

**ZIFA** We downloaded the Python package from https://github.com/epierson9/ZIFA, and used the *fitModel* function with default parameters.

**PPCA-NB** We used the *gllvm* function in the R package **gllvm** (version 1.3.0), and set *method = “VA"*, *Lambda.struc = “diagonal"*, *row.eff = “fixed"*, and *family = “negative.binomial"*.

**pmr** We downloaded it from https://github.com/yuanpeicao/composition-estimate, and used the *autoTuneProxGradient* function with default parameters.

**dmm** We used the *dmn* function in the R package **DirichletMultinomial** (version 1.30.0), with *k=10* Dirichlet components.

**metagenomeSeq** We used the *fitFeatureModel* function in the R package **metagenomeSeq** (version 1.30.0) with default parameters.

**SAVER** We used the *saver* function in the R package **SAVER** (version 1.1.2), and set *ncores = 12*.

**mbImpute** We used the *mbImpute* function in the R package **mbImpute** (version 0.1.0), and set *ncores = 4*.

## Supplementary Information

**Additional file 1** It includes detailed information on simulation experiments, supplementary tables and figures for simulations and empirical data analysis, and the proposed algorithm of variational approximation for ZIPPCA.

**Additional file 2** Review history.

## Data Availability

The software implementing mbDenoise can be downloaded from https://github.com/YanyZeng/mbDenoise [105]. The source code and data for reproducing main figures in the article are available at 10.5281/zenodo.5876795 [106]. We evaluated mbDenoise along with state-of-the-art methods using a collection of published human microbiome datasets (Table 1). Datasets 1, 3, 4, 5, and 6 [33, 66–69] are contained in the R package **curatedMetagenomicData**, and dataset 2 [58] is available at https://microbiomejournal.biomedcentral.com/articles/10.1186/2049-2618-2-32. Dataset 1 [33] is a subset of data from a study that assessed the influence of geography on gut microbiome of healthy individuals. Dataset 2 [58] is from a study linking oral microbiome to chronic periodontitis. And datasets 3-6 [66–69] all involve associating gut microbiome with colorectal cancer, and were also used in validating mbImpute.
