# Author Correction: mbDenoise: microbiome data denoising using zero-inflated probabilistic principal components analysis

**DOI:** 10.1186/s13059-023-02940-x

**Published:** 2023-04-21

**Authors:** Yanyan Zeng, Jing Li, Chaochun Wei, Hongyu Zhao, Tao Wang

**Affiliations:** 1 Department of Bioinformatics and Biostatistics, Shanghai Jiao Tong University, Shanghai, China; 2 Department of Biostatistics, Yale University, New Haven, CT USA; 3 SJTU-Yale Joint Center for Biostatistics and Data Science, Shanghai Jiao Tong University, Shanghai, China; 4 Department of Statistics, School of Mathematical Sciences, Shanghai Jiao Tong University, Shanghai, China; 5 Joint International Research Laboratory of Metabolic & Developmental Sciences, Shanghai Jiao Tong University, Shanghai, China

**Correction: Genome Biol 23, 94 (2022)**

**https://doi.org/10.1186/s13059-022-02657-3**

Following publication of the original article [1], the authors identified an error in the author name of Tao Wang. Their given name and family name were erroneously transposed.

The incorrect author name is: Wang Tao

The correct author name is: Tao Wang

The author group has been updated above and the original article [1] has been corrected.
