# Supplementary material for: mbDenoise: microbiome data denoising using zero-inflated probabilistic principal components analysis
Source: Genome Biol. 2022 Apr 14;23:94. doi: 10.1186/s13059-022-02657-3 (PMC9011970; doi:10.1186/s13059-022-02657-3)
Supplement: Supplementary file 1 — Additional file 1 It includes detailed information on simulation experiments, supplementary tables and figures for simulations and empirical data analysis, and the proposed algorithm of variational approximation for ZIPPCA. [file 13059_2022_2657_MOESM1_ESM.pdf]

SUPPLEMENTARY INFORMATION

mbDenoise: microbiome data denoising using zero-inflated probabilistic principal components analysis

Yanyan Zeng[1], Jing Li[1], Chaochun Wei[1], Hongyu Zhao[2,3*], Tao Wang[1,3,4,5*]

[1]Department of Bioinformatics and Biostatistics, Shanghai Jiao Tong University
[2]Department of Biostatistics, Yale University
[3]SJTU-Yale Joint Center for Biostatistics and Data Science, Shanghai Jiao Tong University
[4]Department of Statistics, Shanghai Jiao Tong University
[5]Joint International Research Laboratory of Metabolic & Developmental Sciences, Shanghai Jiao Tong University

[*]E-mail: hongyu.zhao@yale.edu; neowangtao@sjtu.edu.cn

Contents

1 Detailed information on simulation experiments 2

1.1 Simulation experiments in the main text 2

1.2 Simulation experiments with a covariate 3

1.3 Simulation experiments for DA testing 4

2 Additional results for simulation experiments 5

2.1 mbDenoise ensures the accuracy of estimation and prediction 5

2.1.1 Simulation results for settings with $n > p$ 5

2.1.2 Simulation results for settings with a covariate 6

2.2 mbDenoise produces more reliable estimation of compositions than other methods . 8

2.2.1 Simulation results for settings with $n > p$ 8

2.2.2 Simulation results for settings with a covariate 9

2.3 mbDenoise outperforms other methods in recovering data 11

2.3.1 Simulation results for settings with $n > p$ 11

2.3.2 Simulations for settings with a covariate 12

2.4 mbDenoise empowers DA analysis 14

2.4.1 Simulation results for other DA testing methods 14

2.4.2 Simulation results with data generated from SparseDOSSA 2 14

3 Additional results for empirical data analysis 16

3.1 Negative control of stool microbiomes of one geographical location 16

3.2 Diversity estimation of tongue microbiomes of chronic periodontitis 18

3.3 Additional analysis of stool microbiomes of colorectal cancer 18

3.4 Negative control of stool microbiomes of healthy controls 21

3.5 Comparison of mbDenoise with other DA testing methods 22

4 The proposed algorithm 23

1

Detailed information on simulation experiments

1.1 Simulation experiments in the main text

In example M1, data were generated from the ZIPPCA-NB ($\phi_j > 0$) model in the Methods section of the main text. Example M2 replaced the negative binomial distribution by the Poisson ($\phi_j = 0$). Example M3 assumed a zero-inflated logistic normal multinomial model of the form

$$\begin{aligned}
\text{latent space} \quad & z_{ij} \overset{ind}{\sim} Bern(\eta_{ij}), \\
& f_{i1}, \ldots, f_{ik} \overset{ind}{\sim} N(0,1), \\
\text{parameter space} \quad & \rho_{ij} = \frac{(1-z_{ij})\exp(\beta_{0j} + \boldsymbol{f}_i^\top \boldsymbol{\beta}_j)}{\sum_{l=1}^{p}(1-z_{il})\exp(\beta_{0l} + \boldsymbol{f}_i^\top \boldsymbol{\beta}_l)}, \\
\text{observation space} \quad & \boldsymbol{x}_i \mid \boldsymbol{\rho}_i, W_i \overset{ind}{\sim} MN(\boldsymbol{\rho}_i, W_i),
\end{aligned}$$

where $\boldsymbol{x}_i = (x_{i1}, \ldots, x_{ip})^\top, W_i = \sum_{j=1}^{p} x_{ij}, \boldsymbol{\rho}_i = (\rho_{i1}, \ldots, \rho_{ip})^\top$, and MN denotes the multinomial distribution. In example M4, data were generated from the ZIFA model

$$\begin{aligned}
\text{latent space} \quad & z_{ij} \overset{ind}{\sim} Bern(\eta_{ij}), \\
& f_{i1}, \ldots, f_{ik} \overset{ind}{\sim} N(0,1), \\
\text{parameter space} \quad & \mu_{ij} = \alpha_{i0} + \beta_{0j} + \boldsymbol{f}_i^\top \boldsymbol{\beta}_j, \\
\text{observation space} \quad & \tilde{x}_{ij} \mid \mu_{ij} \overset{ind}{\sim} N(\mu_{ij}, \sigma_j^2), \\
& x_{ij} \mid \tilde{x}_{ij}, z_{ij} = (1-z_{ij})\tilde{x}_{ij}.
\end{aligned}$$

This is a zero-inflated log normal model, whose positive part generates continuous data instead of counts. Example M5 was borrowed from Niku et al, in which data were generated from a negative binomial latent variable model without zero inflation

$$\begin{aligned}
\text{latent space} \quad & \boldsymbol{f}_i \overset{ind}{\sim} p(\boldsymbol{f}_i), \\
\text{parameter space} \quad & \log \mu_{ij} = \alpha_{i0} + \beta_{0j} + \boldsymbol{f}_i^\top \boldsymbol{\beta}_j, \\
\text{observation space} \quad & x_{ij} \mid \mu_{ij} \overset{ind}{\sim} NB(\mu_{ij}, \phi_j).
\end{aligned}$$

Here, $p(\boldsymbol{f}_i) = 0.4N((-1,1)^\top, 0.5\mathbf{I}_2) + 0.3N((2,1.5)^\top, 0.5\mathbf{I}_2) + 0.3N((0.5,-1.5)^\top, 0.5\mathbf{I}_2)$. Table S1.1 provides the model parameters used in the simulation.

Table S1.1: Specification of parameters in M1-M5. W_i/α_{i0} refers to the sequencing depth W_i for M3 or the sample-specific effect α_{i0} for the rest, and σ_j/ϕ_j refers to the standard deviation σ_j for M4 or the overdispersion parameter for the rest.

Example	n	p	k	β_{jr}	β_{0j}	W_i/α_{i0}	σ_j/ϕ_j	η_{ij}
M1	60	100	2	$U(-3,3)$	0	$U(-5,5)$	0.1	0.25
	100	60	2	$U(-3,3)$	0	$U(-5,5)$	0.1	0.25
M2	60	100	2	$U(-3,3)$	0	$U(-5,5)$	0	0.25
	100	60	2	$U(-3,3)$	0	$U(-5,5)$	0	0.25
M3	60	100	2	$U(-1,1)$	0	$U(80,800)$	-	0.25
	100	60	2	$U(-1,1)$	0	$U(50,500)$	-	0.25
M4	50	150	2	$U(-0.5,0.5)$	$U(2.7,3.3)$	0	$U(0.27,0.33)$	$\exp(-0.1\tilde{x}_{ij}^2)$
	150	50	2	$U(-0.5,0.5)$	$U(2.7,3.3)$	0	$U(0.27,0.33)$	$\exp(-0.1\tilde{x}_{ij}^2)$
M5	50	100	2	$U(-2,2)$	$U(-1,1)$	$U(-1,1)$	1	-
	100	50	2	$U(-2,2)$	$U(-1,1)$	$U(-1,1)$	1	-

Finally, in example M6, data were generated from the multinomial model

$$\text{parameter space} \quad W_i = 3np\left(\sum_{i=1}^{n} M_i\right)^{-1} M_i,$$

$$\rho_{ij} = \left(\sum_{j=1}^{p}\sum_{r=1}^{k}|u_{ir}|h_{jr}\right)^{-1}\sum_{r=1}^{k}|u_{ir}|h_{jr},$$

$$\text{observation space} \quad \boldsymbol{x}_i \mid \boldsymbol{\rho}_i, W_i \overset{ind}{\sim} MN\left(\boldsymbol{\rho}_i, W_i\right),$$

where, as in Cao et al, $M_i \overset{ind}{\sim} U(1,10)$, $u_{ir} \overset{ind}{\sim} N(0,1)$, and $h_{jr} \overset{ind}{\sim} I(j \neq r)Bern(0.3) + I(j = r) + N(0,10^{-6})$. We set $(n,p) = (100, 50)$ and $(n,p) = (100, 200)$, each with $k = 20$. To obtain non-negative counts, we repeated the data generating process until ρ_{ij} were all positive.

1.2 Simulation experiments with a covariate

Examples M1-M3 were extended to M7-M9 by setting

$$\log\mu_{ij} = \alpha_{i0} + \beta_{0j} + \gamma_j v_i + \boldsymbol{f}_i^\top\boldsymbol{\beta}_j$$

for M7 and M8 and

$$\rho_{ij} = \frac{(1-z_{ij})\exp(\beta_{0j} + \gamma_j v_i + \boldsymbol{f}_i^\top\boldsymbol{\beta}_j)}{\sum_{l=1}^{p}(1-z_{il})\exp(\beta_{0l} + \gamma_l v_i + \boldsymbol{f}_i^\top\boldsymbol{\beta}_l)}$$

for M9, where v_i is the covariate (e.g., healthy versus diseased) and γ_j is the coefficient. In the same way as in M7 and M8, example M5 was extended to M10. We set

$$v_i = \begin{cases} 1 & i = 1,\ldots,n/2, \\ 0 & i = n/2+1,\ldots,n, \end{cases}$$

for M7-M9, and $v_i \overset{ind}{\sim} N(0,1)$ for M10. Moreover,

$$\gamma_j = \begin{cases} 3 & j = 1,2,\ldots,p/4, \\ -3 & j = p/4+1,p/4+2,\ldots,p/2, \\ 0 & j = p/2+1,p/2+2,\ldots,p, \end{cases}$$

for M7 and M8,

$$\gamma_j = \begin{cases} 0.09 & j = 1, 2, \dots, p/4, \\ -0.09 & j = p/4+1, p/4+2, \dots, p/2, \\ 0 & j = p/2+1, p/2+2, \dots, p, \end{cases}$$

for M9, and $\gamma_j \overset{ind}{\sim} U(-1, 1)$ for M10. Table S1.2 gives the parameters used in the simulation.

Table S1.2: Specification of parameters in M7-M10. W_i/α_{i0} refers to the sequencing depth W_i for M9 or the sample-specific effect α_{i0} for the rest.

Example	n	p	k	β_{jr}	β_{0j}	W_i/α_{i0}	ϕ_j	η_{ij}
M7	60	100	2	$U(-3,3)$	0	$U(-5,5)$	0.1	0.25
	100	60	2	$U(-3,3)$	0	$U(-5,5)$	0.1	0.25
M8	60	100	2	$U(-3,3)$	0	$U(-5,5)$	0	0.25
	100	60	2	$U(-3,3)$	0	$U(-5,5)$	0	0.25
M9	60	100	2	$U(-1,1)$	0	$U(80,800)$	-	0.25
	100	60	2	$U(-1,1)$	0	$U(50,500)$	-	0.25
M10	50	100	2	$U(-2,2)$	$U(-1,1)$	0	1	-
	100	50	2	$U(-2,2)$	$U(-1,1)$	0	1	-

1.3 Simulation experiments for DA testing

Examples M7-M9 with varied γ_j were used in DA analysis. Specifically, we set

$$\gamma_j = \begin{cases} s & j = 1, 2, \dots, p/4, \\ -s & j = p/4+1, p/4+2, \dots, p/2, \\ 0 & j = p/2+1, p/2+2, \dots, p, \end{cases}$$

for M7 and M8, and

$$\gamma_j = \begin{cases} 0.03s & j = 1, 2, \dots, p/4, \\ -0.03s & j = p/4+1, p/4+2, \dots, p/2, \\ 0 & j = p/2+1, p/2+2, \dots, p, \end{cases}$$

for M9, where $s \in \{0, 1, 2, 3, 4, 5\}$ represents the effect size. The rest of the parameters were the same as those in Table S1.2.

2

Additional results for simulation experiments

We present results for settings with $n > p$, and those for settings with a covariate. Note that mbDenoise-zinb-cov, mbDenoise-zip-cov, and PPCA-NB-cov denote the covariate-adjusted version of mbDenoise-zinb, mbDenoise-zip, and PPCA-NB, respectively.

2.1 mbDenoise ensures the accuracy of estimation and prediction

2.1.1 Simulation results for settings with $n > p$

Fig. S2.1 shows estimation and prediction errors for mbDenoise and other methods for settings with $n > p$. We see that the results were similar to those for settings with $n < p$ in the main text.

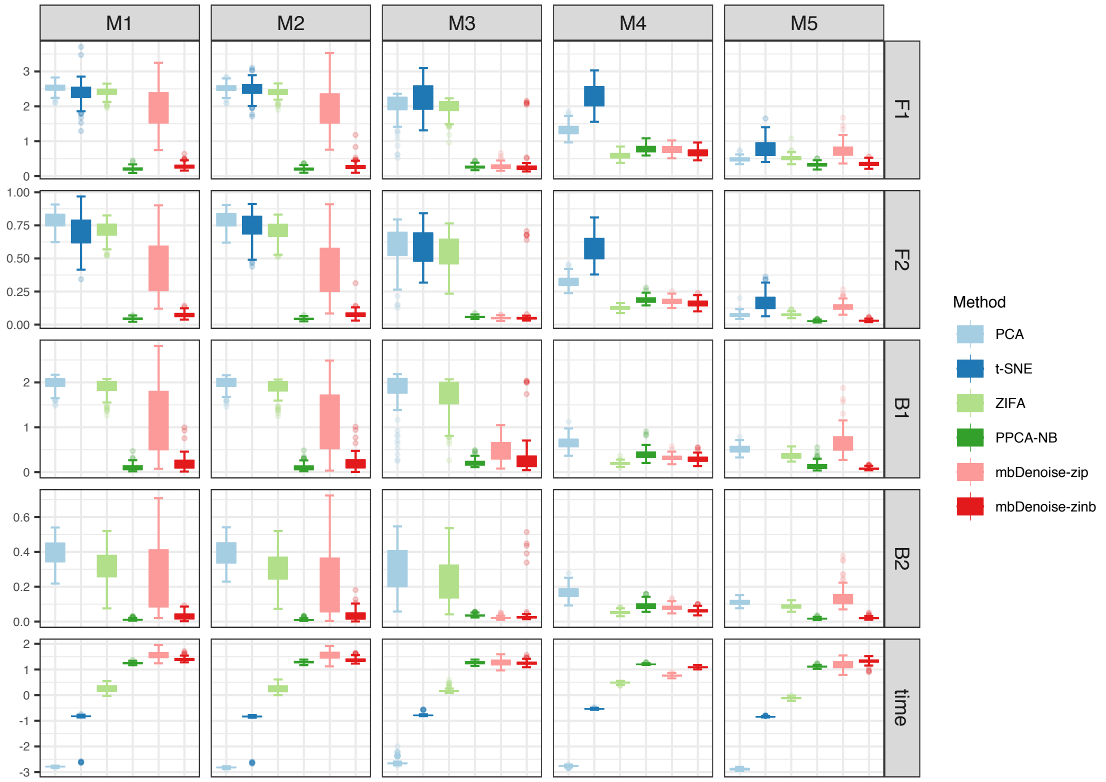

Fig. S2.1: Boxplots of error measures of estimation and prediction for mbDenoise and other methods for settings with $n > p$. F1 and F2 denote the orthogonal projection distance and symmetric Procrustes error for predicting $\boldsymbol{f}_i$, B1 and B2 represent those for estimating $\boldsymbol{\beta}_j$, all averaged over 100 data replications, and time is the average computation time in seconds on the log base 10 scale. Absence of results fort-SNE in B1 and B2 was due to no estimation of $\boldsymbol{\beta}_j$ in t-SNE.

2.1.2 Simulation results for settings with a covariate

Fig. S2.2 and S2.3 show estimation and prediction errors for mbDenoise and other methods for settings with a covariate. We can see that mbDenoise-zinb-cov and PPCA-NB-cov performed well.

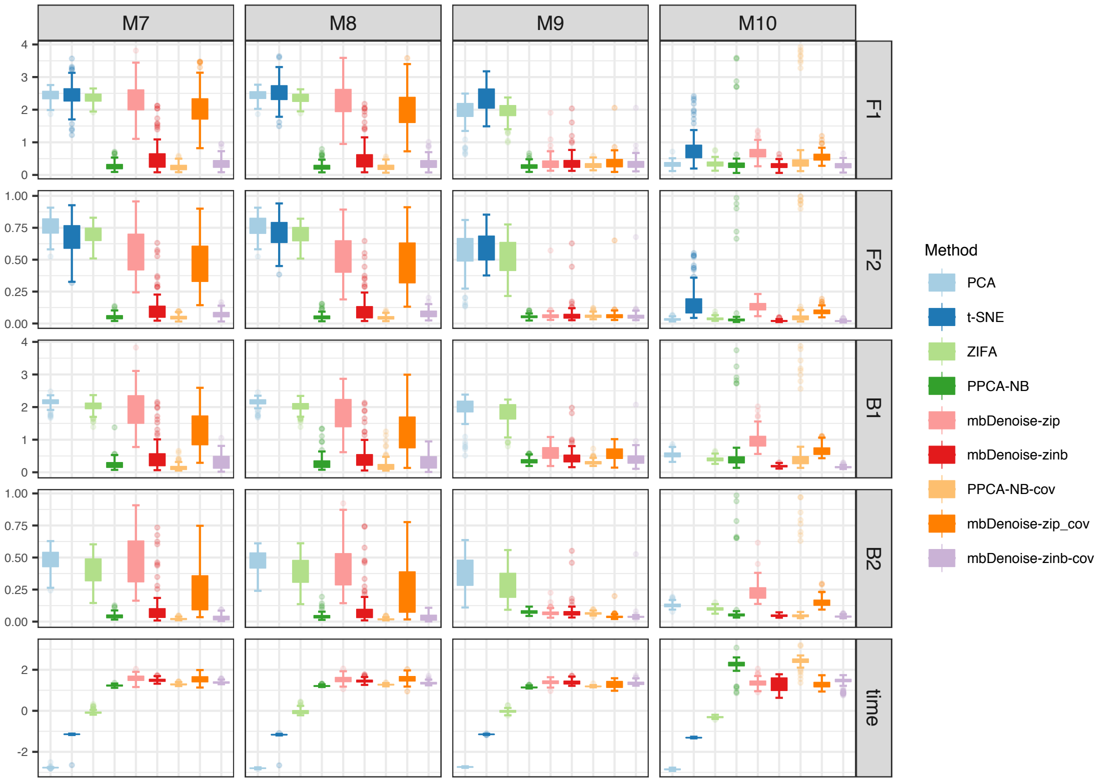

Fig. S2.2: Boxplots of error measures of estimation and prediction for mbDenoise and other methods for settings with a covariate and $n < p$. F1 and F2 denote the orthogonal projection distance and symmetric Procrustes error for predicting $\boldsymbol{f}_i$, B1 and B2 represent those for estimating $\boldsymbol{\beta}_j$, all averaged over 100 data replications, and time is the average computation time in seconds on the log base 10 scale. Absence of results for t-SNE in B1 and B2 was due to no estimation of $\boldsymbol{\beta}_j$ in t-SNE.

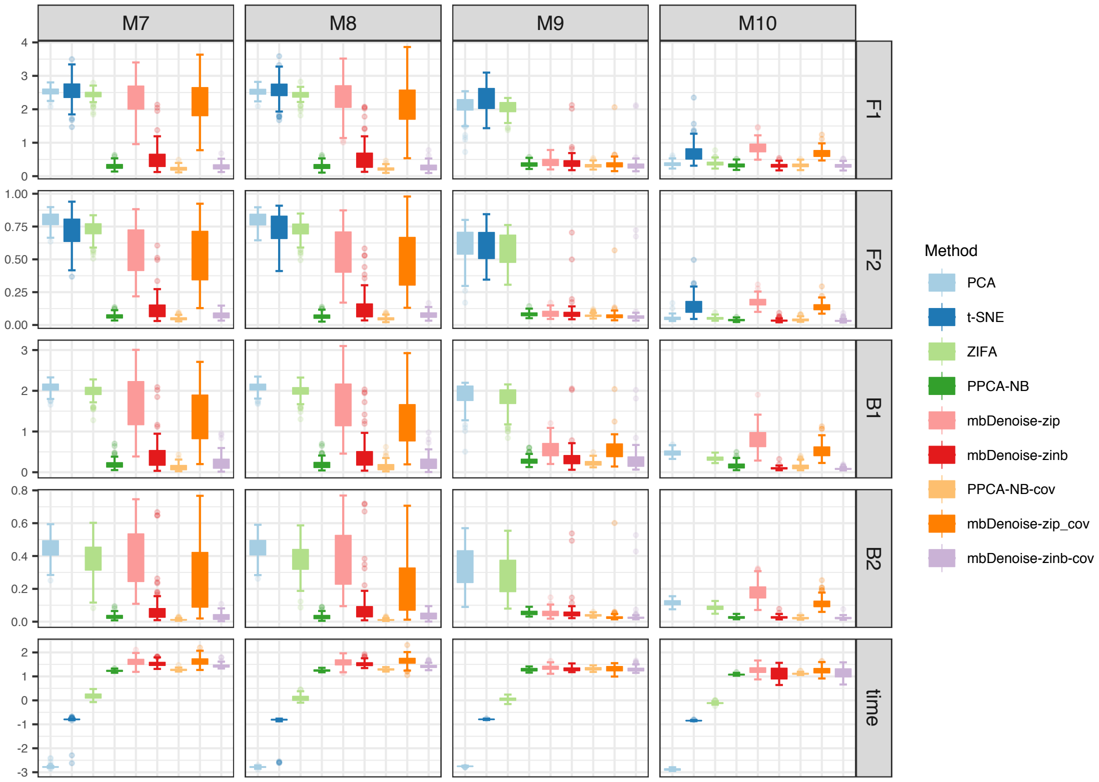

Fig. S2.3: Boxplots of error measures of estimation and prediction for mbDenoise and other methods for settings with a covariate and $n > p$. F1 and F2 denote the orthogonal projection distance and symmetric Procrustes error for predicting $\boldsymbol{f}_i$, B1 and B2 represent those for estimating $\boldsymbol{\beta}_j$, all averaged over 100 data replications, and time is the average computation time in seconds on the log base 10 scale. Absence of results for t-SNE in B1 and B2 was due to no estimation of $\boldsymbol{\beta}_j$ in t-SNE.

2.2 mbDenoise produces more reliable estimation of compositions than other methods

2.2.1 Simulation results for settings with $n > p$

Fig. S2.4 shows errors of composition estimation for mbDenoise and other methods for settings with $n > p$. Again, the results were similar to those for settings with $n < p$ in the main text.

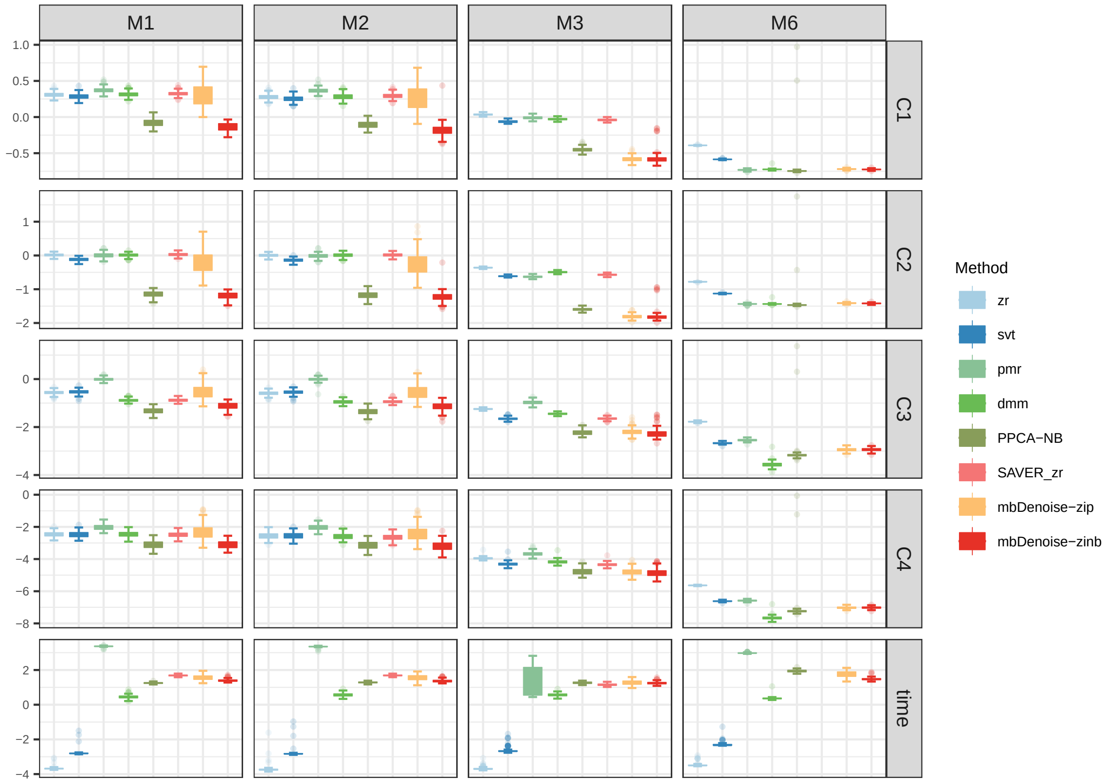

Fig. S2.4: Boxplots for error measures of composition estimation for mbDenoise and other methods for settings with $n > p$. C1-C4 denote the Frobenius norm error, Kullback–Leibler divergence, Shannon's index mean squared error, and Simpson's index mean squared error, and time stands for computation time in seconds, all averaged over 100 data replications and on the log base 10 scale. Absence of results for SAVER_zr in M6 was due to an exception in SAVER.

2.2.2 Simulation results for settings with a covariate

Fig. S2.5 and S2.6 shows errors of composition estimation for mbDenoise and other methods for settings with a covariate. mbDenoise-zinb-cov was clearly the winner.

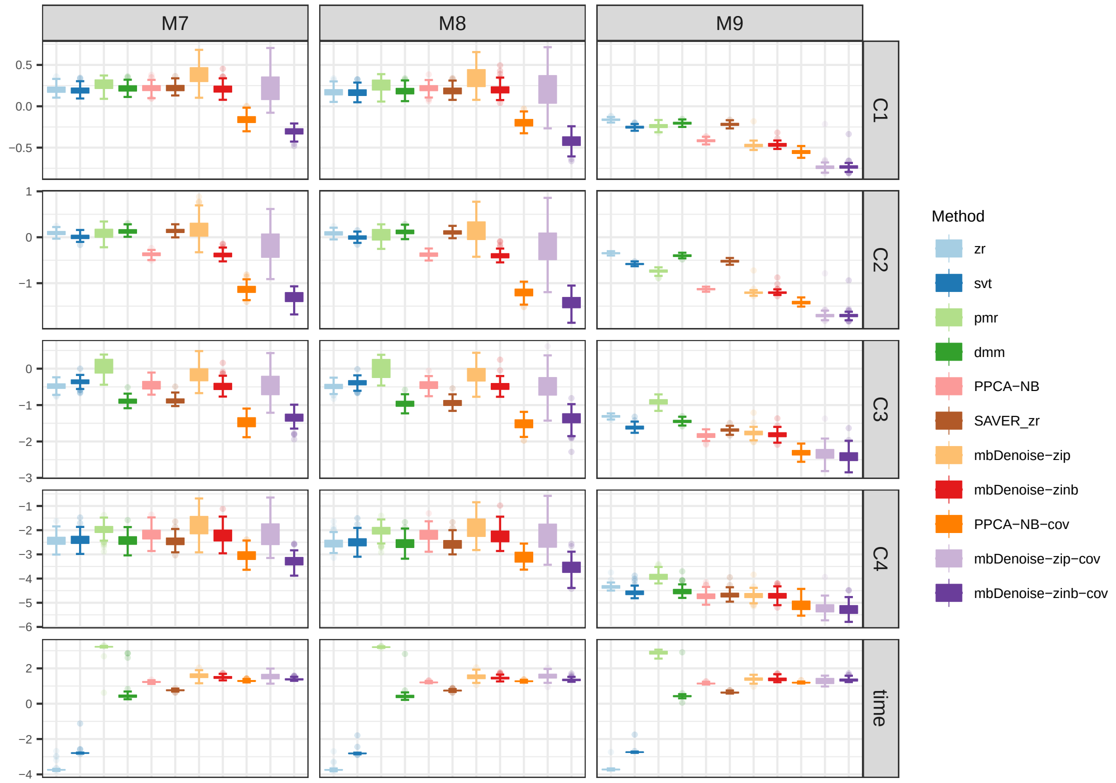

Fig. S2.5: Boxplots for error measures of composition estimation for mbDenoise and other methods for settings with a covariate and $n < p$. C1-C4 denote the Frobenius norm error, Kullback–Leibler divergence, Shannon's index mean squared error, and Simpson's index mean squared error, and time stands for computation time in seconds, all averaged over 100 data replications and on the log base 10 scale.

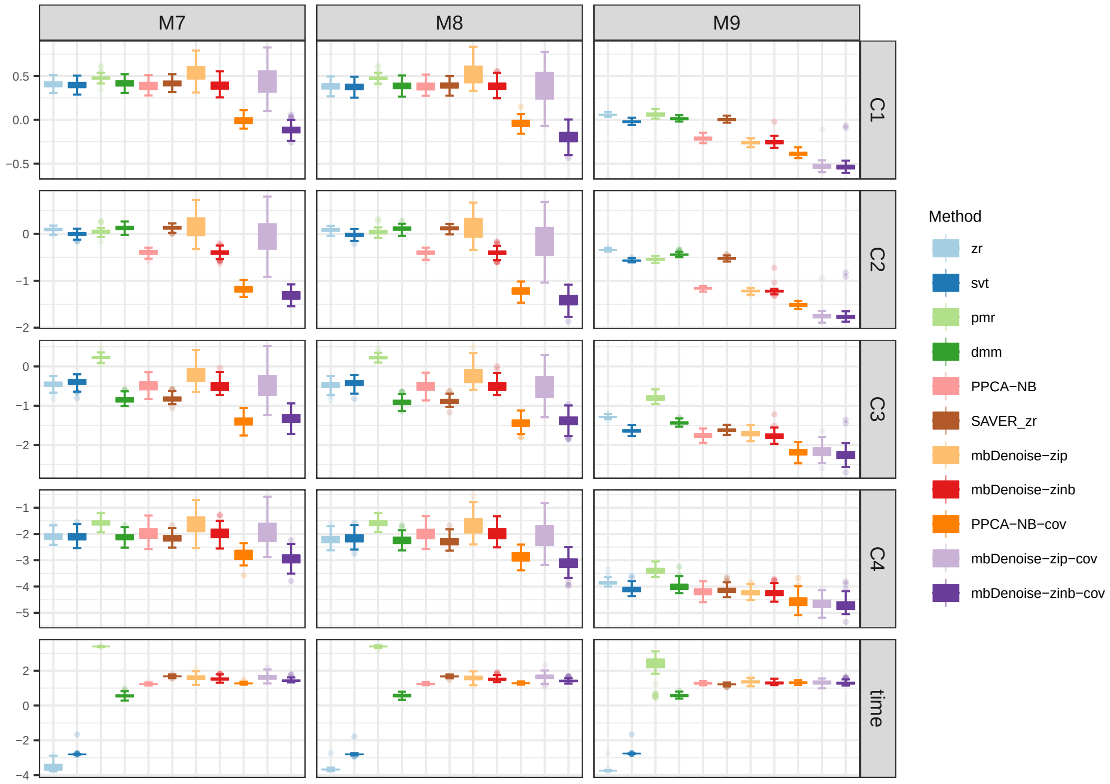

Fig. S2.6: Boxplots for error measures of composition estimation for mbDenoise and other methods for settings with a covariate and $n > p$. C1-C4 denote the Frobenius norm error, Kullback–Leibler divergence, Shannon's index mean squared error, and Simpson's index mean squared error, and time stands for computation time in seconds, all averaged over 100 data replications and on the log base 10 scale. Absence of results for SAVER_zr in M6 was due to an exception in SAVER.

2.3 mbDenoise outperforms other methods in recovering data

2.3.1 Simulation results for settings with $n > p$

Fig. S2.7 shows errors of data recovery for mbDenoise and other methods for settings with $n > p$. We can see that the results were similar to those for settings with $n < p$ in the main text.

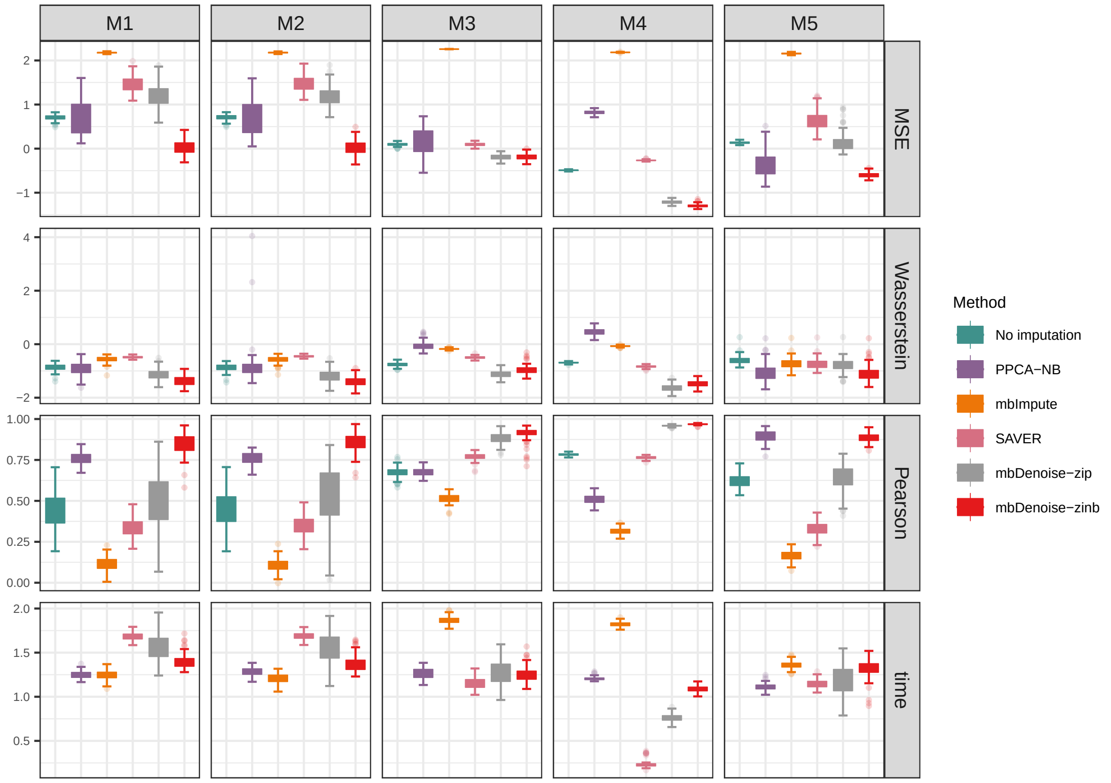

Fig. S2.7: Boxplots for error measures of data recovery for mbDenoise and other methods for settings with $n > p$. MSE and Wasserstein: mean squared error and Wasserstein distance between the denoised matrix and the signal matrix, averaged over 100 data replications and on the log base 10 scale. Pearson: average of Pearson correlation between the denoised and true abundance data. time: average of computation time in seconds on the log base 10 scale.

2.3.2 Simulations for settings with a covariate

Fig. S2.8 and S2.9 show errors of data recovery for mbDenoise and other methods for settings with a covariate. We see that mbDenoise-zinb-cov outperformed the others.

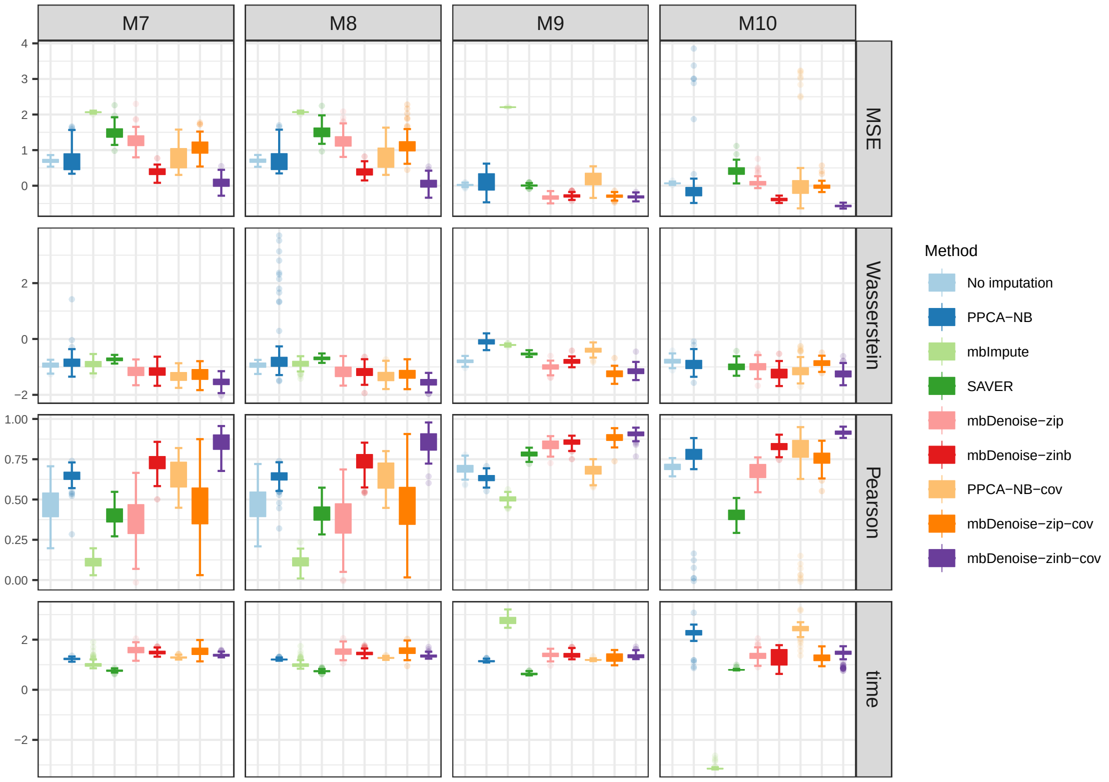

Fig. S2.8: Boxplots for error measures of data recovery for mbDenoise and other methods for settings with a covariate and $n < p$. MSE and Wasserstein: mean squared error and Wasserstein distance between the denoised matrix and the signal matrix, averaged over 100 data replications and on the log base 10 scale. Pearson: average of Pearson correlation between the denoised and true abundance data. time: average of computation time in seconds on the log base 10 scale. Absence of results for mbImpute in M10 was because mbImpute cannot deal with continuous covariates.

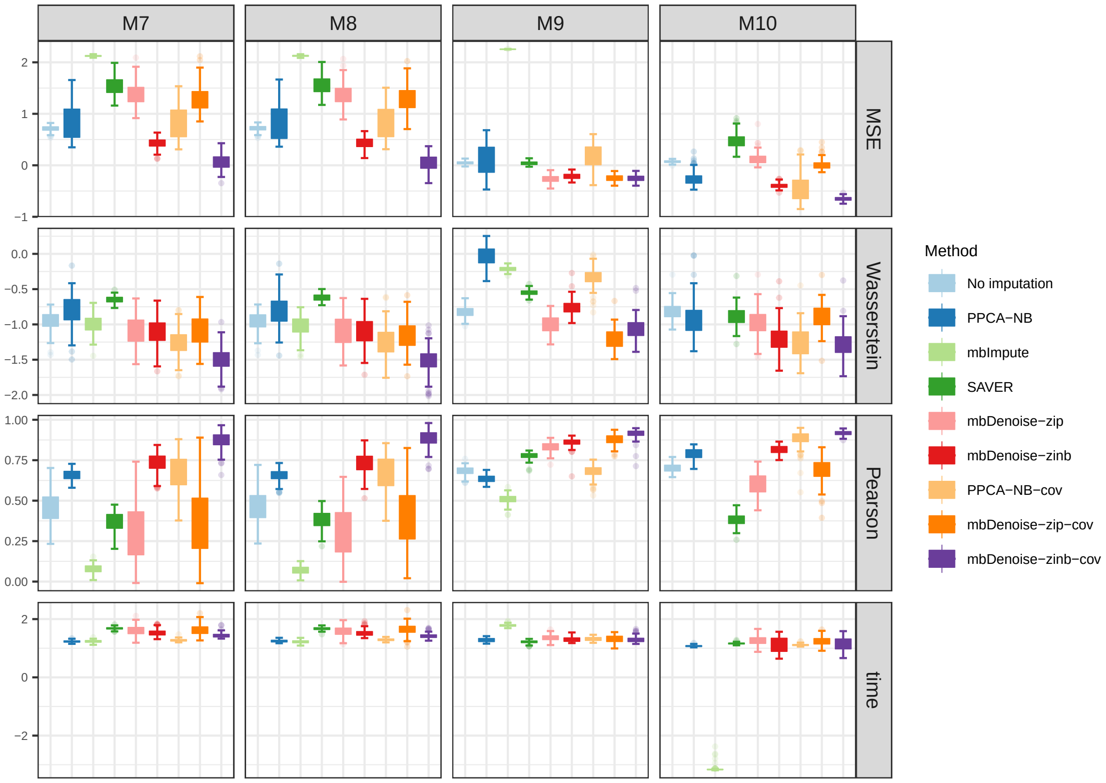

Fig. S2.9: Boxplots for error measures of data recovery for mbDenoise and other methods for settings with a covariate and $n > p$. MSE and Wasserstein: mean squared error and Wasserstein distance between the denoised matrix and the signal matrix, averaged over 100 data replications and on the log base 10 scale. Pearson: average of Pearson correlation between the denoised and true abundance data. time: average of computation time in seconds on the log base 10 scale. Absence of results for mbImpute in M10 was because mbImpute cannot deal with continuous covariates.

2.4 mbDenoise empowers DA analysis

2.4.1 Simulation results for other DA testing methods

From Fig. S2.10, we can see that CSS and TMM performed poorly with very low recall, ZINB had very low precision in M7 and M8, and ANCOM had very low recall in M7-M9. Overall, the proposed method mbDenoise-zinb-cov outperformed ZINB, ANCOM, CSS and TMM.

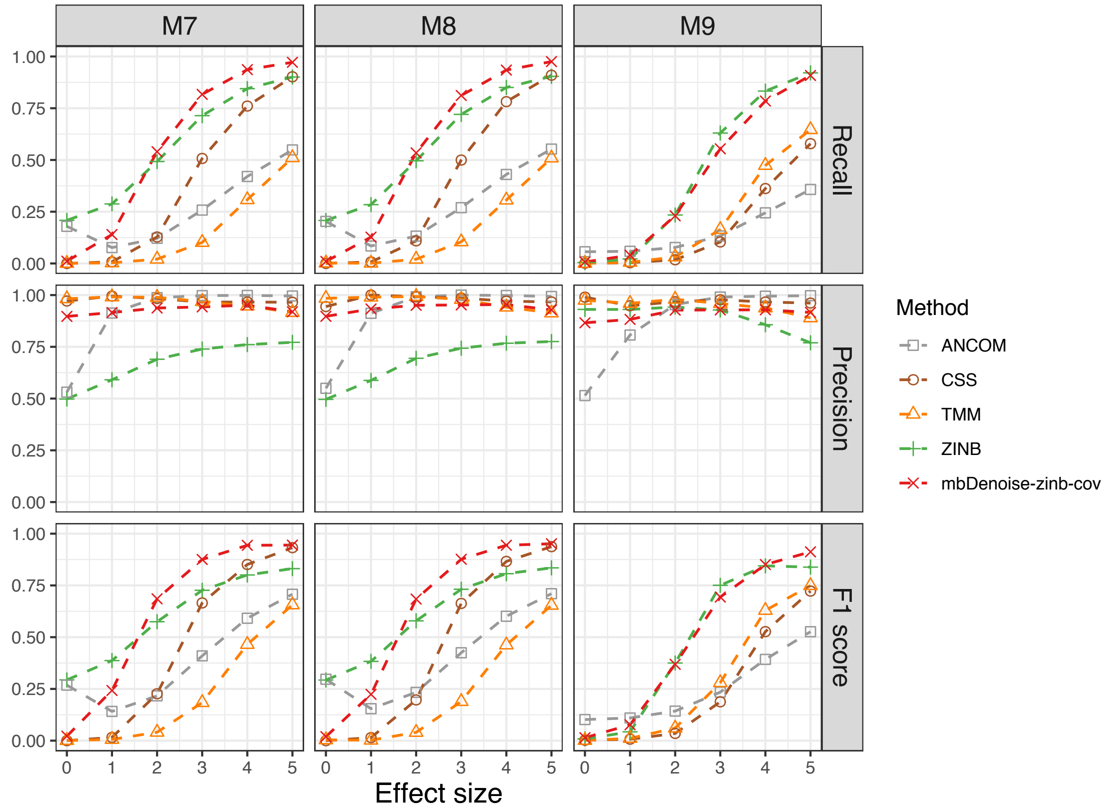

Fig. S2.10: Recall, precision, and F1 score for various testing methods across different effect sizes, each averaged over 100 data replications. We used a FDR threshold of 0.05.

2.4.2 Simulation results with data generated from SparseDOSSA 2

We also applied SparseDOSSA 2 to simulate data for DA analysis. From Table S2.1, we can see that the proposed method mbDenoise-zinb-cov performed well compared to the best that was done in each case.

The Poisson and negative binomial distributions have been widely studied and used for modeling univariate count-valued data. Multivariate generalizations of them that permit rich dependencies, however, have been far less popular, and can be divided into two main classes: (i) where the marginal distributions are Poisson/negative binomial, and (ii) where the joint distribution is a mixture of independent Poisson/negative binomial distributions. Many of the recent developments in the first class use the general copula framework. The sparseDOSSA 2 method also belongs to this class, with some modifications. In particular, it specifies zero-inflated log-normal marginal distributions, invokes normalization to address compositionality, models feature-feature correlations through a multivariate Gaussian copula, and uses a penalized estimation procedure to address high dimensionality. The proposed method mbDenoise belongs to the second class by specifying

Table S2.1: Recall, precision, and F1 score, averaged over 100 data replications, for various testing methods across different effect sizes, when data were generated by SparseDOSSA 2 with built-in vaginal samples as real source data.

	t test	CSS	TMM	ANCOM	edgeR	metagenomeSeq	SAVER	mbImpute	ZINB	mbDenoise-zinb-cov
						(a) Effect size=1				
Recall	0.00	0.00	0.00	0.04	0.16	0.02	0.00	0.31	0.27	0.42
Precision	0.99	0.98	1.00	0.49	0.49	0.97	1.00	0.43	0.44	0.46
F1 score	0.00	0.00	0.00	0.07	0.24	0.04	0.00	0.36	0.33	0.44
						(b) Effect size=2				
Recall	0.01	0.01	0.00	0.03	0.27	0.26	0.01	0.41	0.39	0.59
Precision	0.99	0.99	1.00	0.73	0.60	0.96	0.98	0.46	0.52	0.58
F1 score	0.01	0.02	0.01	0.05	0.37	0.41	0.01	0.43	0.44	0.59
						(c) Effect size=5				
Recall	0.16	0.22	0.12	0.05	0.72	0.67	0.20	0.56	0.66	0.90
Precision	0.87	0.95	0.89	0.90	0.66	0.89	0.86	0.47	0.56	0.63
F1 score	0.28	0.36	0.22	0.09	0.69	0.76	0.33	0.51	0.61	0.74
						(d) Effect size=10				
Recall	0.33	0.39	0.29	0.07	0.88	0.63	0.37	0.66	0.39	0.94
Precision	0.43	0.57	0.58	0.60	0.57	0.63	0.40	0.45	0.30	0.45
F1 score	0.37	0.46	0.39	0.13	0.69	0.63	0.39	0.54	0.34	0.61

zero-inflated negative binomial marginals and accounting for compositionality. The main difference between mbDenoise and sparseDOSSA 2 arises from the way that high dimensionality is addressed. Instead of assuming sparse feature-feature correlations as in sparseDOSSA 2, an alternative is to assume a low rank correlation structure as in mbDenoise. Mixture models are particularly helpful if there is overdispersion in the data, which is often the case for microbiome count data. The ZIPPCA model underlying mbDenoise also belongs to the class of generalized latent variable models, which are increasingly being used for dimension reduction and for studying the factors driving co-occurrence among taxa. Nevertheless, exact inference of the parameters in latent variable models is typically computationally difficult due to the presence of latent mixing variables.

3

Additional results for empirical data analysis

3.1 Negative control of stool microbiomes of one geographical location

We conducted a negative control analysis of Bhopal samples by randomly assigning a binary label (Bhopal/Kerala) to each sample. Ordination analysis and diversity estimation in Fig. S3.1a and S3.1b show that there was no significant difference in community composition between the two groups, for all methods except PPCA-NB. Fig. S3.1c shows that metagenomeSeq, *t*-test, SAVER, and mbDenoise-zinb-cov detected few or no species. By contrast, DESeq2, mbImpute, and egdeR all identified a few species, suggesting that they could not control the false discovery rate.

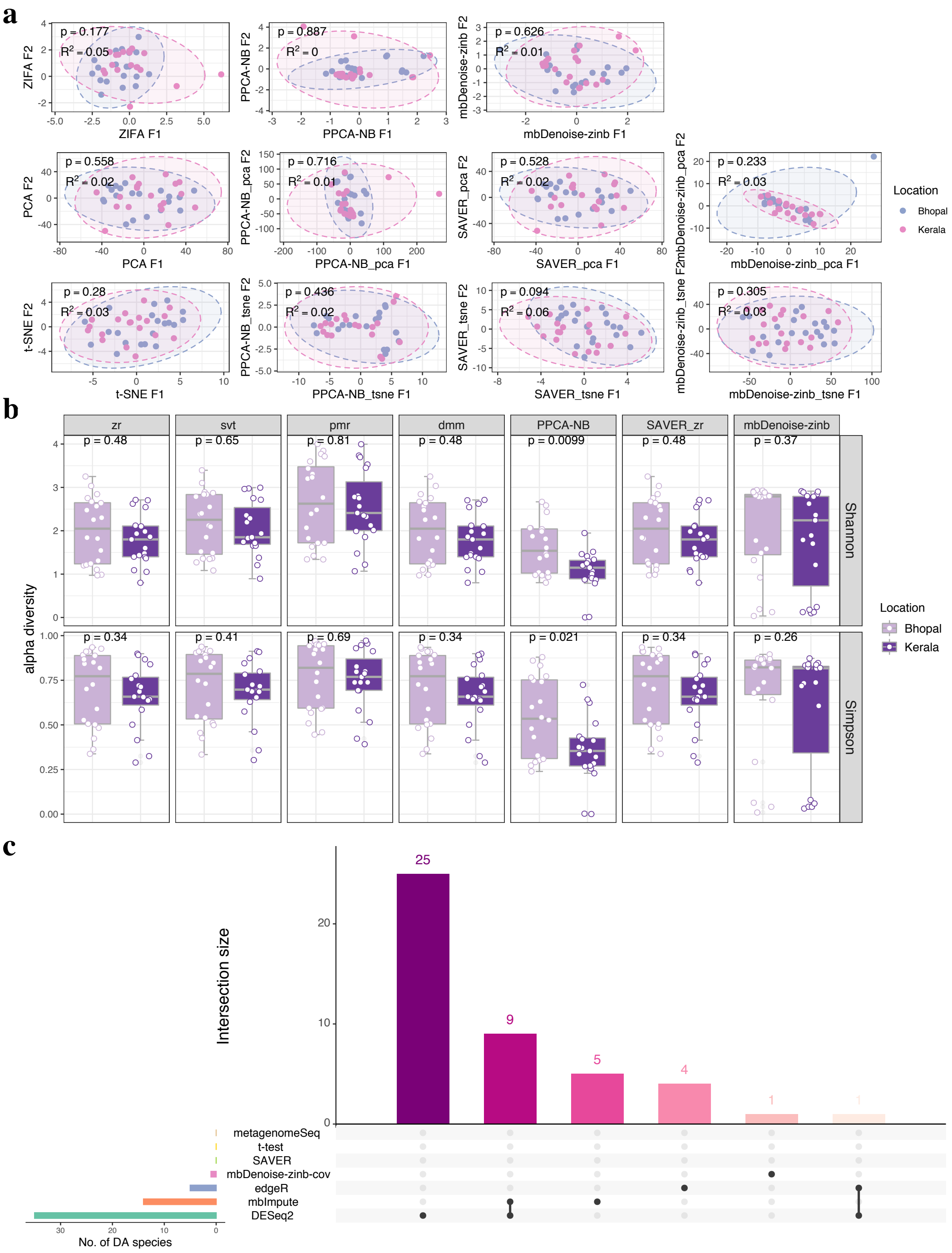

Fig. S3.1: Negative control analysis of stool microbiomes of Bhopal samples. Before carrying out the analysis, each sample was randomly assigned a binary label (Bhopal/Kerala). **a** Data ordination through algorithm-based (PCA and t-SNE), model-based (ZIFA, PPCA-NB, mbDenoise-zinb), and denoising methods (PPCA-NB, mbDenoise-zinb and SAVER) by applying PCA and t-SNE to the denoised data. Inputs of PCA and t-SNE were log-transformed. Beta diversity was assessed using permutational multivariate analysis of variance (PERMANOVA). **b** Alpha diversity analysis. Included methods for composition estimation were zr, svt, pmr, dmm, empirical Bayes estimate by PPCA-NB and by mbDenoise-zinb, and zr using the denoised data from SAVER (SAVER_zr). Significance was calculated using the Wilcoxon test. **c** Visualization of sets of differentially abundant (DA) species between the two groups. Shown are sets detected by *t*-test, DEseq2, edgeR, metagenomeSeq, and *t*-test applied to imputed/denoised data (mbImpute, SAVER, and mbDenoise-zinb-cov). We used a FDR threshold of 0.05.

3.2 Diversity estimation of tongue microbiomes of chronic periodontitis

Fig. S3.2a reveals significant community distinctions between CP and control groups, and between gingiva and tongue, but hardly any difference between subg and supra sites.

We further carried out alpha diversity analysis using tongue samples from CP patients and healthy controls (Fig. S3.2b). Only mbDenoise-zinb revealed that patients with CP had significantly higher alpha diversity than those of control subjects, which is consistent with previous studies.

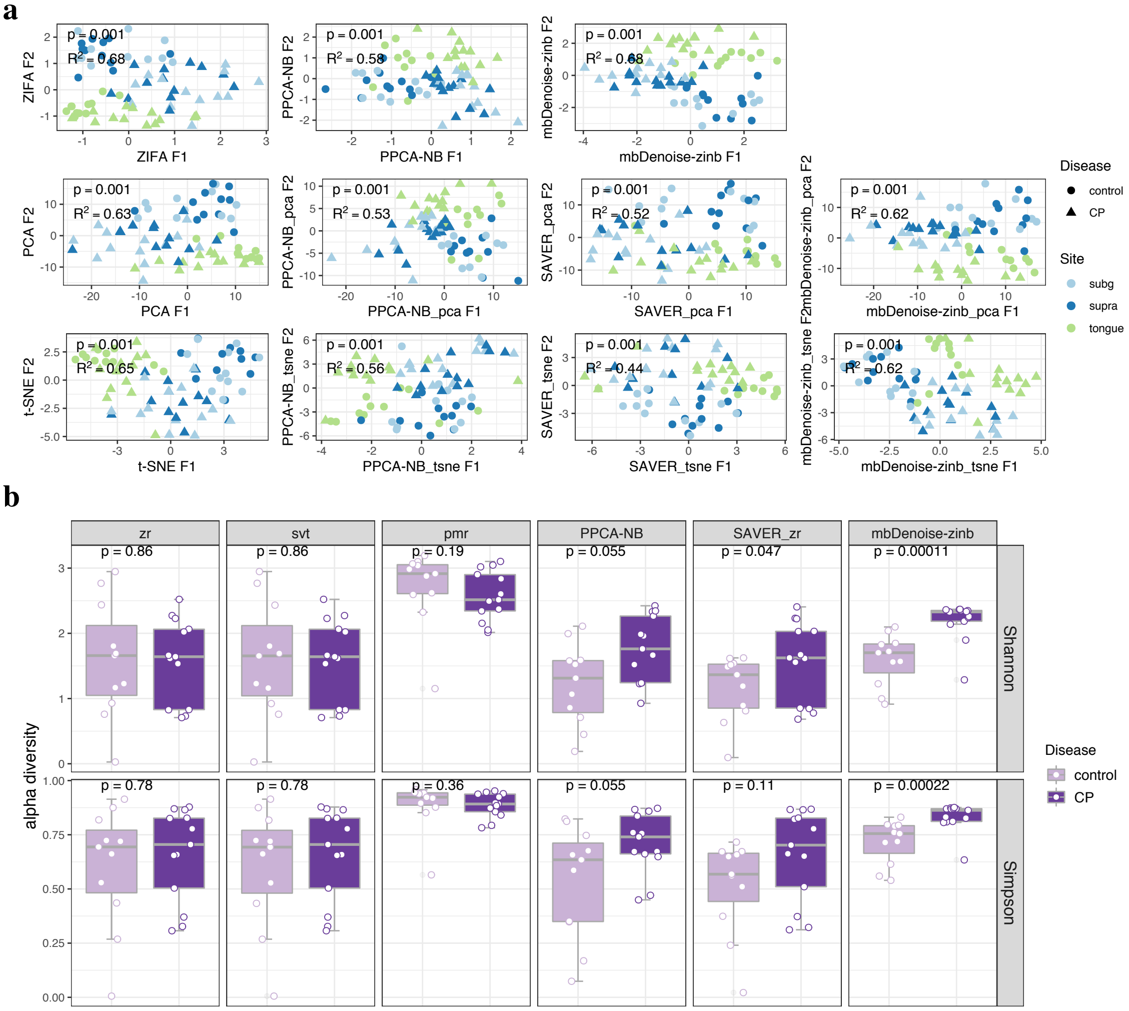

Fig. S3.2: Analysis of oral microbiomes of chronic periodontitis. **a** Data ordination and beta diversity analysis between CP patients and healthy controls, and between gingival (subg and supra) sites and tongue. Included were algorithm-based (PCA and t-SNE), model-based (ZIFA, PPCA-NB, mbDenoise-zinb), and denoising methods (PPCA-NB, mbDenoise-zinb and SAVER) by applying PCA and t-SNE to the denoised data. Inputs of PCA and t-SNE were log-transformed. Beta diversity was assessed using PERMANOVA. **b** Alpha diversity analysis of tongue samples from CP patients and healthy controls. Included methods for composition estimation were zr, svt, pmr, dmm, empirical Bayes estimate by PPCA-NB and by mbDenoise-zinb, and zr using the denoised data from SAVER (SAVER_zr). Significance was calculated using the Wilcoxon test. Absence of results for dmm was due to an exception that disrupts the program execution.

3.3 Additional analysis of stool microbiomes of colorectal cancer

Fig. S3.3 and S3.4 show the results of ordination analysis and diversity estimation for the fourth to sixth datasets in Table 1 in the main text. There was evidence of community dissimilarity between CRC patients and healthy controls (Fig. S3.3a and S3.3b). Furthermore, there was no discernible difference in alpha diversity between the two groups, with the exception of mbDenoise-zinb in the

fifth dataset (Fig. S3.4b).

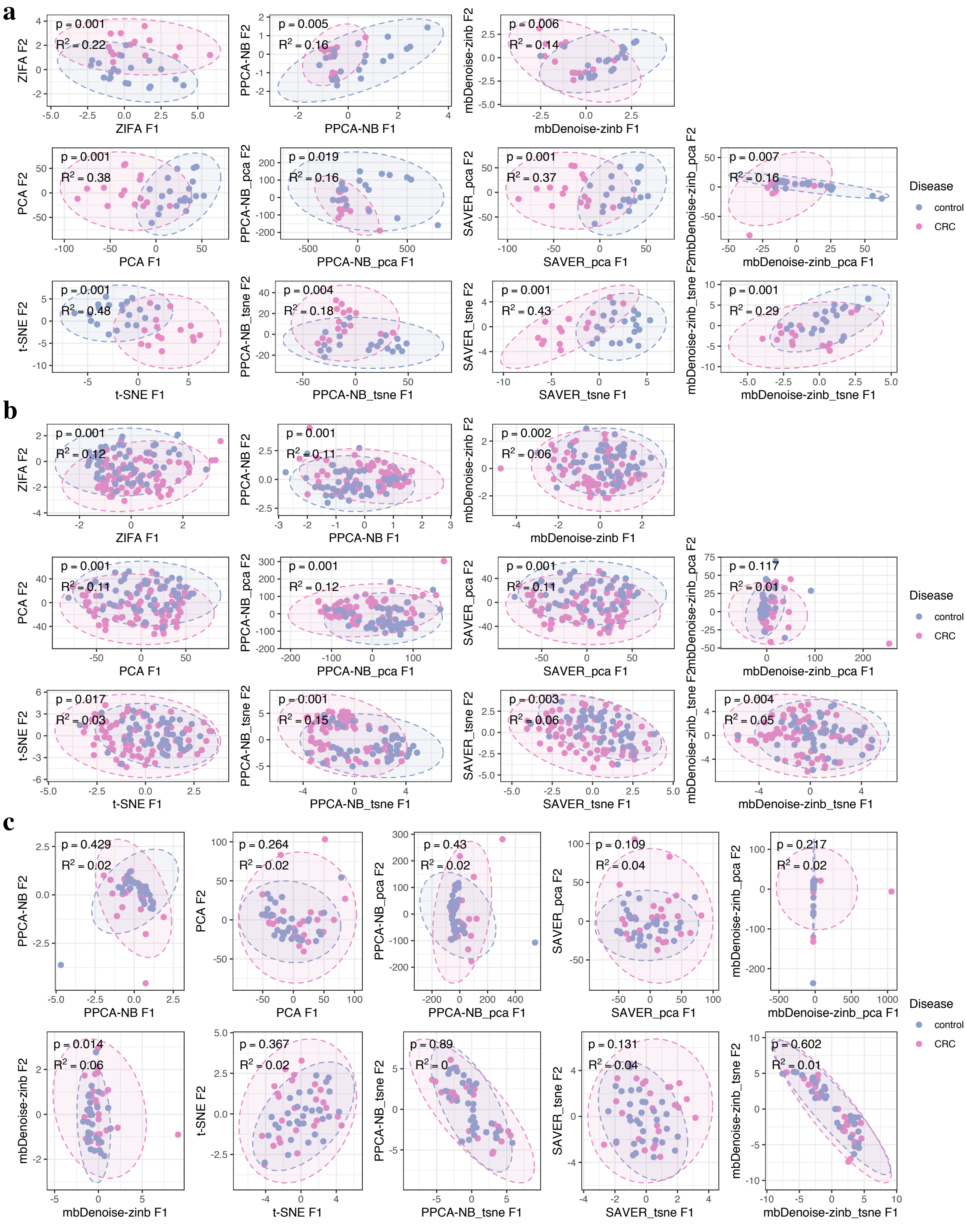

Fig. S3.3: Data ordination and beta diversity analysis between CRC patients and healthy controls **a** on the fourth dataset, **b** on the fifth dataset, and **c** on the sixth dataset in Table 1 in the main text. Included were algorithm-based (PCA and t-SNE), model-based (ZIFA, PPCA-NB, mbDenoise-zinb), and denoising methods (PPCA-NB, mbDenoise-zinb and SAVER) by applying PCA and t-SNE to the denoised data. Inputs of PCA and t-SNE were log-transformed, and the empty was due to an exception in ZIFA. Beta diversity was assessed using PERMANOVA.

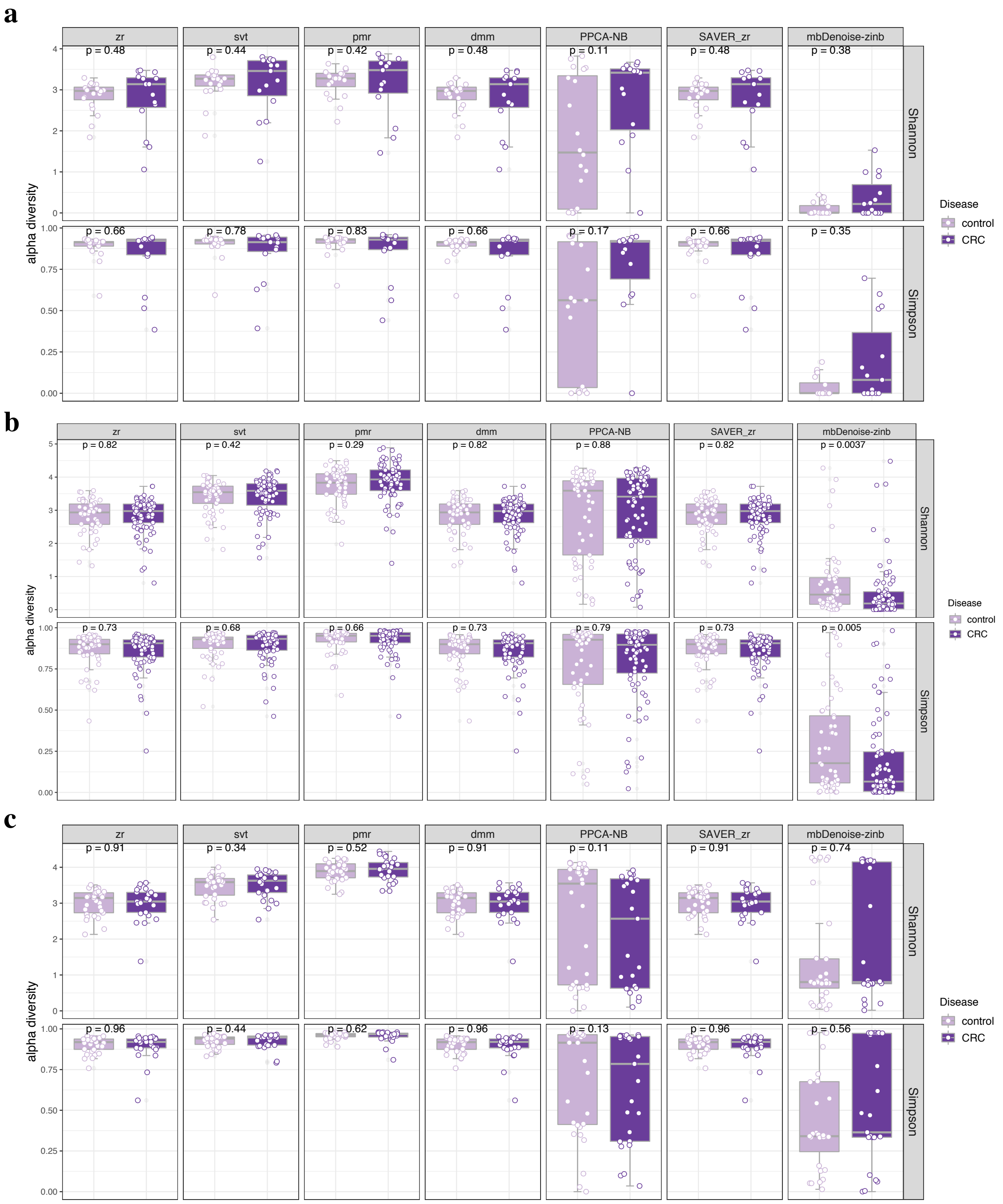

Fig. S3.4: Alpha diversity analysis between CRC patients and healthy controls **a** on the fourth dataset, **b** on the fifth dataset, and **c** on the sixth dataset in Table 1 in the main text. Included methods for composition estimation were zr, svt, pmr, dmm, empirical Bayes estimate by PPCA-NB and by mbDenoise-zinb, and zr using the denoised data from SAVER (SAVER_zr). Significance was calculated using the Wilcoxon test.

3.4 Negative control of stool microbiomes of healthy controls

Again, we conducted a negative control analysis of healthy samples by randomly assigning a binary label (CRC/control) to each sample. Fig. S3.5 shows that the proportions of species declared DA by mbDenoise-zinb-cov were less than those by DESeq2 and mbImpute.

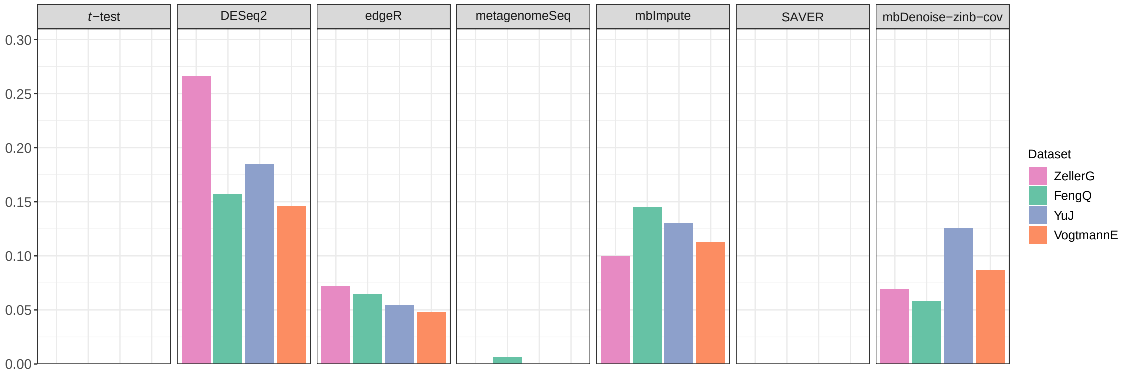

Fig. S3.5: Proportions of species declared as DA by various methods across four datasets, each with CRC samples removed. Before carrying out DA analysis, each healthy sample was randomly assigned a binary label (CRC/control).

3.5 Comparison of mbDenoise with other DA testing methods

We also compared our method with ZINB, ANCOM, CSS and TMM in real data analysis. Fig. S3.6 and S3.7 show that, ZINB was prone to more false discoveries and ANCOM was very conservative. CSS and TMM performed poorly with very low recall, and their performance was similar to that of ANCOM. These were consistent with the findings in the simulation (see Fig. S2.10).

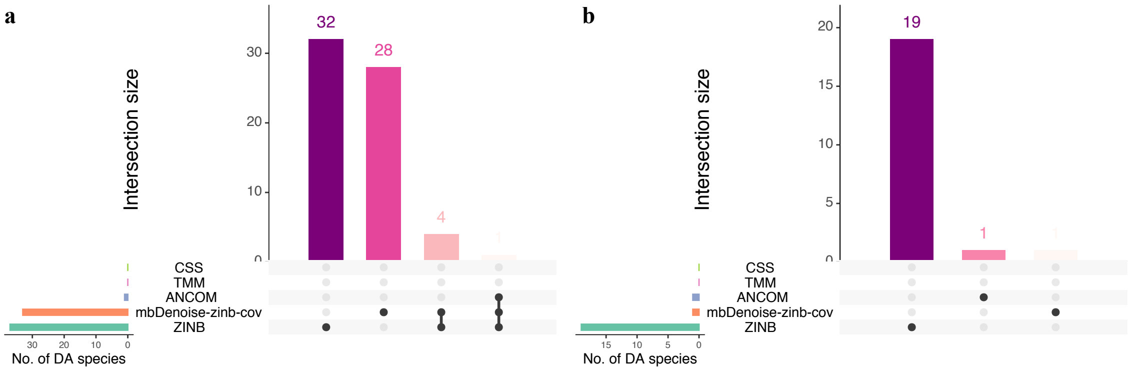

Fig. S3.6: Visualization of sets of differentially abundant (DA) species **a** between Bhopal and Kerala, and **b** on Bhopal samples. Shown are sets detected by ANCOM, ZINB, and *t*-test applied to normalized data by CSS and TMM, and to denoised data by mbDenoise-zinb-cov. We used a FDR threshold of 0.05. Before carrying out DA analysis, each healthy sample in (b) was randomly assigned a binary label (Bhopal/Kerala).

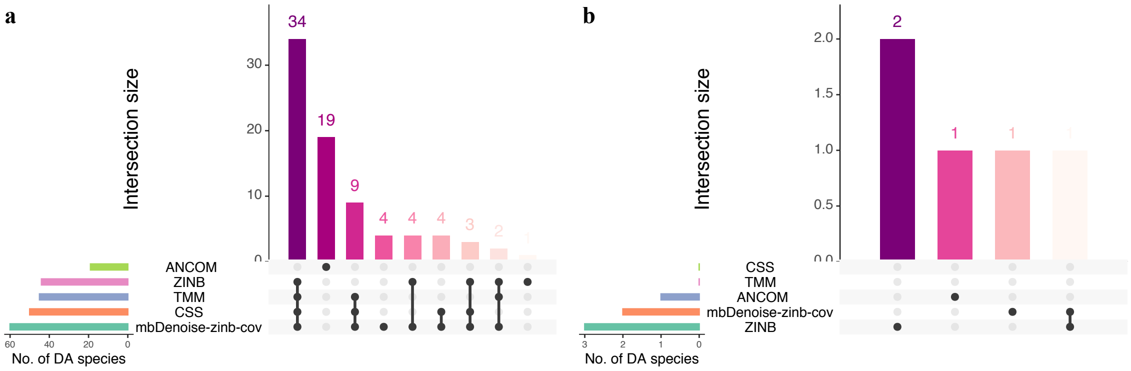

Fig. S3.7: Visualization of sets of differentially abundant (DA) species **a** between CP and control groups, and **b** between subg and supra sites. Shown are sets detected by ANCOM, ZINB, and *t*-test applied to normalized data by CSS and TMM, and to denoised data by mbDenoise-zinb-cov. We used a FDR threshold of 0.05.

4

The proposed algorithm

By replacing the intractable posterior distribution $p(\boldsymbol{f}_i, \boldsymbol{z}_i \mid \boldsymbol{x}_i)$ of the latent variables $(\boldsymbol{f}_i, \boldsymbol{z}_i)$ with a variational famliy of distributions $q(\boldsymbol{f}_i, \boldsymbol{z}_i)$, our VA algorithm can be viewed as a relaxation to EM: estimating the variational parameters amounts to an approximate E-step, while estimating the model parameters corresponds to an approximate M-step.

Thus, we maximize the ELBO by alternating between updating model parameters with variational parameters fixed, and updating variational parameters with model parameters fixed, as outlined in Algorithm 1.

Algorithm 1 Variational Approximation for ZIPPCA

Initialize $\{\tau_j^{(0)}, c_i^{(0)}, \beta_{0j}^{(0)}, \boldsymbol{\beta}_j^{(0)}, \phi_j^{(0)}, \alpha_{i0}^{(0)}, \boldsymbol{m}_i^{(0)}, \boldsymbol{\Sigma}_i^{(0)}, \boldsymbol{\pi}_i^{(0)}\}$, and $s = 0$;
while variational lower bound not converged **do**
    $s = s + 1$;
    given $\{\alpha_{i0}^{(s-1)}, \boldsymbol{m}_i^{(s-1)}, \boldsymbol{\Sigma}_i^{(s-1)}, \boldsymbol{\pi}_i^{(s-1)}\}$, update $\tau_j^{(s)}, c_i^{(s)}, \beta_{0j}^{(s)}, \boldsymbol{\beta}_j^{(s)}$ and $\phi_j^{(s)}$ in turn;
    while parameters not converged **do**
        given $\{\tau_j^{(s)}, c_i^{(s)}, \beta_{0j}^{(s)}, \boldsymbol{\beta}_j^{(s)}, \phi_j^{(s)}\}$, update $\boldsymbol{\pi}_i^{(s)}, \boldsymbol{\Sigma}_i^{(s)}, \alpha_{i0}^{(s)}$ and $\boldsymbol{m}_i^{(s)}$ in turn;
    end while
end while
