# Supplementary material for: mbDenoise: microbiome data denoising using zero-inflated probabilistic principal components analysis
Source: Genome Biol. 2022 Apr 14;23:94. doi: 10.1186/s13059-022-02657-3 (PMC9011970; doi:10.1186/s13059-022-02657-3)
Supplement: Supplementary file 2 — Additional file 2 Review history. [file 13059_2022_2657_MOESM2_ESM.docx]

**1^st^ round**

**Reviewer 1**

The authors proposed to use zero-inflated probabilistic PCA to recover the true abundance levels of taxa. The proposed method is compared with some existing methods and is very promising. The following are my comments and concerns.

For 16S rRNA microbiome data, library size is a frequent confounding factor that obscures biologically meaningful results. It is common that the library sizes vary a lot across the samples. But in the simulation studies and real data analysis, it seems that library size is not explicitly considered. Could you please explain how you handle library size in simulation studies and in real data analysis?

Zero-inflated negative binomial distribution has been popularly used in analyzing microbiome data and detecting differentially abundant taxa since it takes into account of overdispersion and zero inflation. Why did not you consider this method for comparison in differential abundance analysis? Another popularly used method for microbiome data analysis is ANCOM.

Since the proposed method is kind of data normalization, it would also be meaningful if the authors compare the performance of proposed method with other popularly used normalization methods for microbiome data including rarefying, proportion, and CSS.

Could you please further explain how gene functional redundancy affects the analysis on taxa abundance? I completely agree that for microbiome data, it is very common that the sample size is relatively small comparing with the number of taxa. But how does "This data redundancy can be addressed by a low-rank approximation" (page 2) work? The latent variables f_i may need further explanation.

**Reviewer 2**

This paper proposes a method for "denoising" microbiome data that entails fitting a hierarchical model in which the observed counts are assumed to come from a zero-inflated negative binomial (ZINB) distribution. The log of the negative binomial mean parameter is then modeled as a linear combination of sample-specific and latent factors, which are constructed using PCA. The authors propose that the "denoised" values obtained as model predictions can be used for a variety of downstream analyses (ordination, diversity, and differential abundance). The paper is generally well-written and contains a number of simulation scenarios and comparators.

Major comments:

- I'm concerned that some of the results seem to reflect that signal is getting washed out of the data. For example, in Figure 6a, the results for "mbDenoise-zinb_pca" (which I think is the proposed method, although there are quite a few variants so it is confusing), many of the samples are aligned along a single gradient, suggesting that variation among these samples has been reduced down to one dimension. Figure 6b shows that for many samples, the "denoised" estimate of alpha diversity is at or very close to zero. This suggests that all information has been washed out of the sample through the denoising.

- While this paper introduces some new ideas for microbiome data denoising (i.e., the use of PCA to construct the latent factors), there are also many similarities between this paper and a recent publication by an overlapping set of authors (i.e., the multi-level zero-inflated negative binomial model and variational inference approach): "MZINBVA: variational approximation for multilevel zero-inflated negative-binomial models for association analysis in microbiome surveys" https://doi.org/10.1093/bib/bbab443 It seems this model can also be used for differential abundance testing. Please clarify and cite this work.

- The authors note that "data redundancy", i.e., the fact that many taxa have similar genes and may perform similar function is a challenge of microbiome data. A common approach to handle this aspect of the data is to take into account phylogenetic tree information. For example, the Unifrac distance metrics (i.e., unweighted, weighted, and generalized Unifrac) incorporate tree information and can be used in ordination and distance-based testing approaches such as PERMANOVA. The simulation designs and the methods considered as comparators in the current paper don't consider the structural relationships among microbiome features.

- The figures need to be streamlined and the results for the proposed method need to be highlighted in some way. See for example Figure 8, which contains an overwhelming number of subfigures.

Minor comments:

- The simulation scenarios are all model-based, and there is some debate about the extent to which such set-ups capture the structure of real microbiome data. There are newer simulation approaches (e.g., SparseDOSSA https://doi.org/10.1371/journal.pcbi.1008913 and SparseDOSSA 2) which may generate more realistic data.

- Bottom of p. 4 - "inputs were log counts" - how were zeros handled? Did you first add a pseudocount?

- Bray-Curtis distance is designed to be applied to count data. A different distance metric might be more appropriate for log-transformed or denoised values.

- The description of the variational algorithm is rather terse - please include somewhat more detail including the definition of the ELBO.

- Is the VA algorithm adopted over EM purely for computational reasons? Do you obtain any estimates of uncertainty for the model parameters using this approach?

- The citations given on microbial network inference in the discussion are old. There is more recent work on microbial network inference using hierarchical models - https://doi.org/10.3389/fgene.2020.00445 and <https://doi.org/10.1080/10618600.2021.1935971>

**Reviewer 3**

This paper presents a parametric latent variable model for modeling microbiome countsdata that includes many zeros. While similar ideas/models have appeared in many other papers (e.g. Sohn and Li, Xu, Demmer and Li), this paper includes very thorough evaluations and comparisons with many different methods in several aspects of microbiome data analysis, where the data sets are generated based on different models. I also found the real data analysis results convincing. I have some minor comments that I would like the authors to address or comment on:

1. It would be helpful to have a more detailed explanation why the authors choose their models instead of those by Sohn and Li, and Xu, Demmer and Li. My sense is that all these three model are based on some latent variable models.

2. Variational approximation is used to fit the proposed models. Since the models are fully parametric, I would think some types of likelihood/quasi-likelihood estimation can be developed that may provide better estimate of the model parameters.

3. I like the negative control experiments that you performed using real data analysis. The authors can make stronger statements about the inappropriateness of using DE-seq and other methods developed for single-cell RNA data for analyzing the microbiome count data. It would also be helpful if you can point out why the methods such as SAVER or DE-seq or edge-R do not work for differential abundance analysis.

4. For DA analysis shown in Fig 5, do you have a way to obtain the statistical significance? It is probably hard since variational approximation is used for estimating the model parameters.

**Responses to Referee 1**

• *The authors proposed to use zero-inflated probabilistic PCA to recover the true abundance*

*levels of taxa. The proposed method is compared with some existing methods*

*and is very promising.*

**Reply:** Thank you very much for your summary and positive assessment of our work.

• *Comments:*

1. *For 16S rRNA microbiome data, library size is a frequent confounding factor that*

*obscures biologically meaningful results. It is common that the library sizes vary*

*a lot across the samples. But in the simulation studies and real data analysis, it*

*seems that library size is not explicitly considered. Could you please explain how*

*you handle library size in simulation studies and in real data analysis?*

**Reply:** Thank you very much for your careful reading and question. To handle

the uneven library sizes across samples, sample-specific parameters *_i*0 are

introduced in the ZIPPCA model so that

log *_ij* = *_i*0 + *_*0*j* + ***f****⊤*

*i* ***_****j :*

We clarify this in the revised manuscript. In the simulation studies, we took the

library size into account, see Tables S1.1 and S1.2 in the supplementary. As the

library size is a known confounding factor, the sample-specific effects are removed

in the denoised data to eliminate the bias caused by library size, as was done in

real data analysis.

2. *Zero-inflated negative binomial distribution has been popularly used in analyzing*

*microbiome data and detecting differentially abundant taxa since it takes into*

*account of overdispersion and zero inflation. Why did not you consider this*

*method for comparison in differential abundance analysis? Another popularly*

*used method for microbiome data analysis is ANCOM.*

**Reply:** Thank you very much for your suggestion. We now include the method

of applying the zero-inflated negative binomial model (denoted by ZINB, Zeileis

et al. 2008) and ANCOM (Mandal et al., 2015) for comparison in differential

abundance analysis in the revised manuscript.

First, we applied ZINB and ANCOM to simulated data, from examples M7-M9,

using the same setup as in Figure 5 in the main text. From Figure 1 (Figure S2.10

in the supplementary), we can see that ZINB had very low precision in M7 and

M8, and ANCOM had very low recall in M7-M9. Overall, the proposed method

mbDenoise-zinb-cov outperformed ZINB and ANCOM.

We also compared our method with ZINB and ANCOM in real data analysis.

Figures 2 and 3 (Figures S3.6-S3.7 in the supplementary) show that, ZINB was

prone to more false discoveries and ANCOM was very conservative. These were

consistent with the findings in the simulation.

3. *Since the proposed method is kind of data normalization, it would also be meaningful*

*if the authors compare the performance of proposed method with other popularly*

*used normalization methods for microbiome data including rarefying, proportion,*

*and CSS.*

**Reply:** Thank you very much for your suggestion. We now compare our denoising

method with two popular normalization methods, cumulative-sum scaling (CSS,

M7 M8 M9

Recall Precision F1 score

0 1 2 3 4 5 0 1 2 3 4 5 0 1 2 3 4 5

0.00

0.25

0.50

0.75

1.00

0.00

0.25

0.50

0.75

1.00

0.00

0.25

0.50

0.75

1.00

Effect size

Method

ANCOM

CSS

TMM

ZINB

mbDenoise-zinb-cov

Figure 1: Recall, precision, and F1 score for various testing methods across different effect sizes,

each averaged over 100 data replications. We used a FDR threshold of 0.05. Full details of the data

generation are given in the supplementary.

6

32

28

4

1

0

10

20

30

Intersection size

ZINB

mbDenoise-zinb-cov

ANCOM

TMM

CSS

30 20 10 0

No. of DA species

19

1 1

0

5

10

15

20

Intersection size

ZINB

mbDenoise-zinb-cov

ANCOM

TMM

CSS

15 10 5 0

No. of DA species

**a b**

Figure 2: Visualization of sets of differentially abundant (DA) species (a) between Bhopal and

Kerala, and (b) on Bhopal samples. Shown are sets detected by ANCOM, ZINB, and *t*-test applied

to normalized data by CSS and TMM, and to denoised data by mbDenoise-zinb-cov. We used a

FDR threshold of 0.05. Before carrying out DA analysis, each healthy sample in (b) was randomly

assigned a binary label (Bhopal/Kerala).

34

19

9

4 4 4 3 2 1

0

10

20

30

Intersection size

mbDenoise-zinb-cov

CSS

TMM

ZINB

ANCOM

60 40 20 0

No. of DA species

2

1 1 1

0.0

0.5

1.0

1.5

2.0

Intersection size

ZINB

mbDenoise-zinb-cov

ANCOM

TMM

CSS

3 21 0

No. of DA species

**a b**

Figure 3: Visualization of sets of differentially abundant (DA) species (a) between CP and control

groups, and (b) between subg and supra sites. Shown are sets detected by ANCOM, ZINB, and

*t*-test applied to normalized data by CSS and TMM, and to denoised data by mbDenoise-zinb-cov.

We used a FDR threshold of 0.05.

Paulson et al. 2013) and trimmed mean of M-values (TMM, Robinson et al. 2010),

in the revised manuscript. For simplicity, we restrict our attention to differential

abundance analysis. For the sake of fairness, we apply Welch’s t-test to the log

transformed normalized/denoised data.

We compared CSS and TMM with mbDenoise-zinb-cov, ZINB, and ANCOM,

using the same simulated and real datasets as in the reply to your comment 2.

The results are again shown in Figures 1-3. We see that CSS and TMM performed

poorly on simulated data with very low recall, and their performance on real data

was similar to that of ANCOM. Hence, in terms of differential abundance testing,

mbDenoise-zinb-cov performed better than CSS and TMM.

4. *Could you please further explain how gene functional redundancy affects the analysis*

*on taxa abundance? I completely agree that for microbiome data, it is very*

*common that the sample size is relatively small comparing with the number of*

*taxa. But how does “This data redundancy can be addressed by a low-rank approximation”*

*(page 2) work? The latent variables* ***f****i may need further explanation.*

**Reply:** Thank you very much for your suggestion. By functional redundancy

we mean that some taxa perform similar functions in communities and ecosystems,

and redundant taxa may therefore be substitutable with little impact on

ecosystem processes (Lawton and Brown, 1994).

In the Methods section of the revised manuscript, we now mention that the lowrank

representation formed by ***f****⊤*

*i* ***_****j* , that is, the linear combinations of latent

factors ***f*** *i* in the linear predictor, takes advantage of the redundancy in microbiome

data. The latent variables ***f*** *i* also induce correlation between taxa, and their

number controls model complexity, and it is helpful to think of them as resembling

the axes in an ordination, representing either missing predictors or the main axes

of covariation of abundance across taxa (Warton et al., 2015).

**Responses to Referee 2**

• *This paper proposes a method for “denoising” microbiome data that entails fitting a*

*hierarchical model in which the observed counts are assumed to come from a zeroinflated*

*negative binomial (ZINB) distribution. The log of the negative binomial mean*

*parameter is then modeled as a linear combination of sample-specific and latent factors,*

*which are constructed using PCA. The authors propose that the “denoised” values*

*obtained as model predictions can be used for a variety of downstream analyses (ordination,*

*diversity, and differential abundance). The paper is generally well-written*

*and contains a number of simulation scenarios and comparators.*

**Reply:** Thank you very much for your summary and positive assessment of our work.

• *Major comments:*

1. *I’m concerned that some of the results seem to reflect that signal is getting washed*

*out of the data. For example, in Figure 6a, the results for “mbDenoise-zinb_pca”*

*(which I think is the proposed method, although there are quite a few variants so it*

*is confusing), many of the samples are aligned along a single gradient, suggesting*

*that variation among these samples has been reduced down to one dimension.*

*Figure 6b shows that for many samples, the “denoised” estimate of alpha diversity*

*is at or very close to zero. This suggests that all information has been washed*

*out of the sample through the denoising.*

**Reply:** Thank you very much for your insightful comment. Within the ZIPPCA

framework, we can use the posterior mean or mode of ***f*** *i* as the ordination

score. Alternatively, we can first denoise the abundance data, and then apply

an algorithm-based method to the denoised data to compute ordination axes. In

this manuscript, we prefer the second strategy, because it retains the versatility

of algorithm-based methods. As the name suggests, mbDenoise-zinb_pca and

mbDenoise-zinb_tsne are proposed for linear and nonlinear dimension reduction,

respectively. For real data sets, the truth is often unknown and the patterns are

often not linear, and hence we recommend mbDenoise-zinb_tsne. From Figure 6a

we see that mbDenoise-zinb_tsne behaved normally. In the revised manuscript,

we make clear the above statement (mbDenoise-zinb_pcoa is deleted, see the

reply to your minor comment 3).

On the other hand, we totally agree that one can never rule out the possibility

that signal may be lost from the data during the denoising step. This is likely

a consequence of the linearity nature of the ZIPPCA model, that is, log *_ij* =

*_i*0 + *_*0*j* + ***f****⊤*

*i* ***_****j :* In practice, the assumption of linearity is questionable and

nonlinear functions of ***f*** *i* such as neural networks might do a better job. We

mention this over-denoising issue as well as nonlinear extensions of the ZIPPCA

framework in the discussion of revised manuscript.

2. *While this paper introduces some new ideas for microbiome data denoising (i.e.,*

*the use of PCA to construct the latent factors), there are also many similarities*

*between this paper and a recent publication by an overlapping set of authors (i.e.,*

*the multi-level zero-inflated negative binomial model and variational inference*

*approach): “MZINBVA: variational approximation for multilevel zero-inflated*

*negative-binomial models for association analysis in microbiome surveys” https:*

9

*//doi.org/10.1093/bib/bbab443. It seems this model can also be used for differential*

*abundance testing. Please clarify and cite this work.*

**Reply:** Thank you very much for your comment. Although both MZINBVA

and mbDenoise use variational approximation for approximate estimation and

inference, there are important differences between these two methods. First,

mbDenoise is a general statistical method for describing and simulating microbial

community profiles in a cross-sectional study, whereas MZINBVA focuses on

differential abundance testing in longitudinal/multi-level studies. Second, mbDenoise

fits the data at the overall community level with rich dependencies among

taxa and independence among samples, whereas MZINBVA fits the data at the

individual taxon level accounting for structured correlations between samples but

ignoring correlations between taxa. In the revised manuscript, we cite this work

and highlight the similarities and differences between MZINBVA and mbDenoise.

In addition, we include the method of applying the zero-inflated negative binomial

model to cross-sectional data (denoted by ZINB, Zeileis et al. 2008) for comparison

in differential abundance analysis; see the reply to comment 2 of Referee 1

for details. Work is in progress to extend mbDenoise to longitudinal/multi-level

designs.

3. *The authors note that “data redundancy”, i.e., the fact that many taxa have similar*

*genes and may perform similar function is a challenge of microbiome data.*

*A common approach to handle this aspect of the data is to take into account*

*phylogenetic tree information. For example, the Unifrac distance metrics (i.e.,*

*unweighted, weighted, and generalized Unifrac) incorporate tree information and*

*can be used in ordination and distance-based testing approaches such as PERMANOVA.*

*The simulation designs and the methods considered as comparators in*

*the current paper don’t consider the structural relationships among microbiome*

*features.*

**Reply:** Thank you very much for raising this interesting and important issue.

mbDenoise is a latent variable modeling approach for denoising microbiome data.

It borrows information across samples and taxa to decouple biological signal from

technical variation. There are two reasons why we do not consider the phylogenetic

relationships among microbes. First, incorporating the tree structure into

the ZIPPCA model underlying mbDenoise is not the same as in the Unifrac and

will inevitably make the current modeling and fitting too complicated. Second, in

practice the phylogeny is inferred from molecular sequences, and so it is necessary

to quantify uncertainty in phylogenetic inference and its impact on downstream

analyses. One possible way to address this problem is to consider the structural

relationships among taxa while carrying out downstream statistical analyses on

the denoised data. Nevertheless, it is interesting yet challenging to describe and

simulate microbial community profiles while taking into account the phylogenetic

tree information. To our knowledge, there are no generally accepted methods

available in the literature. We add a discussion on this issue in the discussion of

the revised manuscript.

4. *The figures need to be streamlined and the results for the proposed method need*

*to be highlighted in some way. See for example Figure 8, which contains an*

*overwhelming number of subfigures.*

**Reply:** Thank you very much for your helpful suggestions. In the revised

manuscript, to improve the readability, we have streamlined the figures and made

clear statements about the performance of the proposed method.

• *Minor comments:*

1. *The simulation scenarios are all model-based, and there is some debate about the*

*extent to which such set-ups capture the structure of real microbiome data. There*

*are newer simulation approaches (e.g., SparseDOSSA https://doi.org/10.1371/jo*

*urnal.pcbi.1008913 and SparseDOSSA 2) which may generate more realistic data.*

**Reply:** Thank you very much for your suggestion. In the revised manuscript,

we now apply sparseDOSSA 2 (Ma et al., 2021) to simulate data for differential

abundance (DA) analysis. We used the built-in vaginal samples as real source

data for sparseDOSSA 2. For DA testing, we set the sample size for each group

*n* = 100 and the number of taxa *p* was by default 109. The fraction of DA

features was 40%. Four values of the effect size (1, 2, 5 and 10) were explored.

From Table 1 (Table S2.1 in the supplementary), we can see that the proposed

method mbDenoise-zinb-cov performed well compared to the best that was done

in each case. Also included for comparison here are CSS, TMM, ANCOM, and

ZINB; see the reply to comments 2 and 3 of Referee 1 for more details.

The Poisson and negative binomial distributions have been widely studied and

Table 1: Recall, precision, and F1 score, averaged over 100 data replications, for various testing

methods across different effect sizes, when data were generated by SparseDOSSA 2 with built-in

vaginal samples as real source data.

t test CSS TMM ANCOM edgeR metagenomeSeq SAVER mbImpute ZINB mbDenoise-zinb-cov

(a) Effect size=1

Recall 0.00 0.00 0.00 0.04 0.16 0.02 0.00 0.31 0.27 0.42

Precision 0.99 0.98 1.00 0.49 0.49 0.97 1.00 0.43 0.44 0.46

F1 score 0.00 0.00 0.00 0.07 0.24 0.04 0.00 0.36 0.33 0.44

(b) Effect size=2

Recall 0.01 0.01 0.00 0.03 0.27 0.26 0.01 0.41 0.39 0.59

Precision 0.99 0.99 1.00 0.73 0.60 0.96 0.98 0.46 0.52 0.58

F1 score 0.01 0.02 0.01 0.05 0.37 0.41 0.01 0.43 0.44 0.59

(c) Effect size=5

Recall 0.16 0.22 0.12 0.05 0.72 0.67 0.20 0.56 0.66 0.90

Precision 0.87 0.95 0.89 0.90 0.66 0.89 0.86 0.47 0.56 0.63

F1 score 0.28 0.36 0.22 0.09 0.69 0.76 0.33 0.51 0.61 0.74

(d) Effect size=10

Recall 0.33 0.39 0.29 0.07 0.88 0.63 0.37 0.66 0.39 0.94

Precision 0.43 0.57 0.58 0.60 0.57 0.63 0.40 0.45 0.30 0.45

F1 score 0.37 0.46 0.39 0.13 0.69 0.63 0.39 0.54 0.34 0.61

used for modeling univariate count-valued data. Multivariate generalizations of

them that permit rich dependencies, however, have been far less popular, and

can be divided into two main classes: (i) where the marginal distributions are

Poisson/negative binomial, and (ii) where the joint distribution is a mixture of

independent Poisson/negative binomial distributions. Many of the recent developments

in the first class use the general copula framework. The sparseDOSSA

2 method also belongs to this class, with some modifications. In particular, it

specifies zero-inflated log-normal marginal distributions, invokes normalization to

address compositionality, models feature-feature correlations through a multivariate

Gaussian copula, and uses a penalized estimation procedure to address high

dimensionality. The proposed method mbDenoise belongs to the second class by

specifying zero-inflated negative binomial marginals and accounting for compositionality.

The main difference between mbDenoise and sparseDOSSA 2 arises

from the way that high dimensionality is addressed. Instead of assuming sparse

feature-feature correlations as in sparseDOSSA 2, an alternative is to assume a

low rank correlation structure as in mbDenoise. Mixture models are particularly

helpful if there is overdispersion in the data, which is often the case for microbiome

count data. The ZIPPCA model underlying mbDenoise also belongs to the

class of generalized latent variable models, which are increasingly being used for

dimension reduction and for studying the factors driving co-occurrence among

taxa (Warton et al., 2015). Nevertheless, exact inference of the parameters in

latent variable models is typically computationally difficult due to the presence

of latent mixing variables (see the reply to your minor comment 5).

2. *Bottom of page 4 - “inputs were log counts” - how were zeros handled? Did you*

*first add a pseudocount?*

**Reply:** Thank you very much for your careful reading. We now clarify that

inputs were log2(1+counts) for PCA, t-SNE, and ZIFA.

3. *Bray-Curtis distance is designed to be applied to count data. A different distance*

*metric might be more appropriate for log-transformed or denoised values.*

**Reply:** Thank you very much for your comment. We now clarify that for methods

whose inputs are log-transformed values, the Euclidean distance was applied.

Note that, using the Euclidean distance, PCoA reduces to PCA. To avoid confusion,

we have deleted PCoA in the revised manuscript.

4. *The description of the variational algorithm is rather terse - please include somewhat*

*more detail including the definition of the ELBO.*

**Reply:** Thank you very much for your suggestion. In the revised manuscript, we

now elaborate on the variational approximation algorithm. Specifically, consider

a variational family of distributions *q*(***f*** *i;* ***z****i*) for the latent variables (***f*** *i;* ***z****i*). Using

Jensen’s inequality, the data log-likelihood satisfies

Σ*n*

*i*=1

log *p*(***x****i*) =

Σ*n*

*i*=1

log

[

*Eq*

{

*p*(***x****i;* ***f*** *i;* ***z****i*)

*q*(***f*** *i;* ***z****i*)

}]

*_Eq*

Σ*n*

*i*=1

*f*log *p*(***x****i;* ***f*** *i;* ***z****i*)*g 􀀀*

Σ*n*

*i*=1

*Eqf*log *q*(***f*** *i;* ***z****i*)*g:*

The right-hand size above is called an evidence lower bound (ELBO). It is easy

to see that maximizing the ELBO with respect to *q*(***f*** *i;* ***z****i*) is equivalent to minimizing

the Kullback–Leibler divergence between *q*(***f*** *i;* ***z****i*) and the true posterior

*p*(***f*** *i;* ***z****i j* ***x****i*). Details on the numerical optimization procedure can be found in

the supplementary.

5. *Is the VA algorithm adopted over EM purely for computational reasons? Do you*

*obtain any estimates of uncertainty for the model parameters using this approach?*

**Reply:** Thank you very much for your comment. Since the integrals involved

in the data likelihood do not have closed form expressions, it is difficult to find

the maximum likelihood estimate. A general technique in the latent variable

modeling literature is the Monte Carlo EM algorithm. However, Monte Carlo

methods are computationally intensive and have mainly been used for small-scale

problems. VA is an attractive non-Monte Carlo alternative. Rather than using

sampling, the main idea behind VA is to use optimization; see the reply to your

minor comment 4.

However, inference using VA is challenging. Nevertheless, some progress has been

made in the literature. For example, Westling and McCormick (2019) build a connection

between VA and profile *M*-estimation, and provide a sandwich covariance

formula for the VA; see also Liu et al. (2021). In the revised manuscript, we add

a discussion on estimation of uncertainty under the VA framework.

6. *The citations given on microbial network inference in the discussion are old. There*

*is more recent work on microbial network inference using hierarchical models - ht*

*tps://doi.org/10.3389/fgene.2020.00445 and https://doi.org/10.1080/10618600.2021.*

*1935971*

**Reply:** Thank you very much for reminding us of these two papers (Jiang et al.,

2020; Osborne et al., 2021) on microbial network inference using hierarchical

models. We now cite them in the discussion of the revised manuscript.

**Responses to Referee 3**

• *This paper presents a parametric latent variable model for modeling microbiome counts*

*data that includes many zeros. While similar ideas/models have appeared in many*

*other papers (e.g. Sohn and Li, Xu, Demmer and Li), this paper includes very*

*thorough evaluations and comparisons with many different methods in several aspects*

*of microbiome data analysis, where the data sets are generated based on different*

*models. I also found the real data analysis results convincing.*

**Reply:** Thank you very much for your summary and positive assessment of our work.

• *Minor comments:*

1. *It would be helpful to have a more detailed explanation why the authors choose*

*their models instead of those by Sohn and Li, and Xu, Demmer and Li. My sense*

*is that all these three models are based on some latent variable models.*

**Reply:** Thank you very much for your comment. Loosely speaking, the proposed

ZIPPCA model and those of Xu et al. (2021) and Sohn and Li (2017) all belong

to the general class of generalized latent variable models. However, the latter

two methods treat the ***f*** *i*’s as unknown parameters to be estimated rather than

random variables. Another difference concerns the underlying count distribution.

It is well-known that the negative binomial distribution in ZIPPCA is preferable

to Poisson (Xu et al., 2021) or quasi-Poisson (Sohn and Li, 2017) in terms of

addressing the overdispersion of microbiome count data. Nevertheless, there is

a price to be paid for the increased flexibility of ZIPPCA, it requires computationally

intensive numerical optimization techniques (see the reply to your minor

comment 2). In the revised manuscript, we now cite these two papers and explain

the similarities and differences between the three models.

2. *Variational approximation is used to fit the proposed models. Since the models are*

*fully parametric, I would think some types of likelihood/quasi-likelihood estimation*

*can be developed that may provide better estimate of the model parameters.*

**Reply:** Thank you very much for your comment. The proposed ZIPPCA model

is fully parametric. However, since the latent variables ***f*** *i* are random rather

than fixed, the integrals involved in the data likelihood do not have closed form

expressions. Consequently, it is difficult to find the maximum likelihood estimate.

A general technique in the latent variable modeling literature is the Monte

Carlo expectation maximization algorithm. However, Monte Carlo methods are

computationally intensive and have mainly been used for small-scale problems.

An alternative is to use the Laplace approximation or penalized quasi-likelihood.

However, compared with variational approximation, the Laplace approximation

is known to suffer from convergence problems (Hui et al., 2017). We add a discussion

on parameter estimation in the revised manuscript.

3. *I like the negative control experiments that you performed using real data analysis.*

*The authors can make stronger statements about the inappropriateness of using*

*DESeq and other methods developed for single-cell RNA data for analyzing the*

*microbiome count data. It would also be helpful if you can point out why the*

*methods such as SAVER or DESeq or edgeR do not work for differential abundance*

*analysis.*

**Reply:** Thank you very much for your suggestions. It has been reported in

the literature that DESeq2 and edgeR both have unexpectedly high false discovery

rates for detecting differentially abundant taxa (Weiss et al., 2017) and for

identifying differentially expressed genes (Li et al., 2021). This is largely due to

their built-in normalization processes (see the reply to comment 3 of referee 1).

On the other hand, SAVER treats all zeros equally and removes extremely lowabundance

features at the beginning, and hence it fails to distinguish technical

zeros from biological zeros, leading to inaccurate abundance/expression estimates

that obscure biologically meaningful results. We now make stronger statements

about the inappropriateness/failure of these methods in the revised manuscript.

4. *For DA analysis shown in Fig 5, do you have a way to obtain the statistical significance?*

*It is probably hard since variational approximation is used for estimating*

*the model parameters.*

**Reply:** Thank you very much for raising this interesting problem. The problem

of inference using variational approximation is challenging. Nevertheless, some

progress has been made in the literature. Assume that the dimension of the units

of analysis tends to infinity, but that the other dimension of features is fixed,

Westling and McCormick (2019) build a connection between variational approximation

and profile *M*-estimation, and provide a sandwich covariance formula for

the variational approximation estimate; see also Liu et al. (2021). However, due

to the complicated nature of the problem, developing a general theory in high dimensions

would be a substantial undertaking that the result would effectively be

a separate paper. Work along this line is in progress. In the revised manuscript,

we now provide a discussion on the limits of our methodology from a theoretical

aspect.

**References**

Hui, F. K., Warton, D. I., Ormerod, J. T., Haapaniemi, V., and Taskinen, S. (2017). Variational

approximations for generalized linear latent variable models. *Journal of Computational*

*and Graphical Statistics*, 26(1):35–43.

Jiang, S., Xiao, G., Koh, A. Y., Chen, Y., and Zhan, X. (2020). HARMONIES: a hybrid

approach for microbiome networks inference via exploiting sparsity. *Frontiers in Genetics*,

11:445.

Lawton, J. H. and Brown, V. K. (1994). *Redundancy in Ecosystems*. Springer, Berlin

Heidelberg.

Li, Y., Ge, X., Peng, F., Li, W., and Li, J. J. (2021). A large-sample crisis? exaggerated

false positives by popular differential expression methods. Preprint at *bioRxiv*

https://doi.org/10.1101/2021.08.25.457733.

Liu, T., Xu, P., Du, Y., Lu, H., Zhao, H., and Wang, T. (2021). MZINBVA:

variational approximation for multilevel zero-inflated negative-binomial models

for association analysis in microbiome surveys. *Briefings in Bioinformatics*.

https://doi.org/10.1093/bib/bbab443.

Ma, S., Ren, B., Mallick, H., Moon, Y. S., Schwager, E., Maharjan, S., et al. (2021).

A statistical model for describing and simulating microbial community profiles. *PLOS*

*Computational Biology*, 17(9):e1008913.

Mandal, S., Van Treuren, W., White, R. A., Eggesbø, M., Knight, R., and Peddada, S. D.

(2015). Analysis of composition of microbiomes: a novel method for studying microbial

composition. *Microbial Ecology in Health and Disease*, 26(1):27663.

16

17

Osborne, N., Peterson, C. B., and Vannucci, M. (2021). Latent network estimation and

variable selection for compositional data via variational EM. *Journal of Computational*

*and Graphical Statistics*. https://doi.org/10.1080/10618600.2021.1935971.

Paulson, J. N., Stine, O. C., Bravo, H. C., and Pop, M. (2013). Differential abundance

analysis for microbial marker-gene surveys. *Nature Methods*, 10(12):1200–1202.

Robinson, M. D., McCarthy, D. J., and Smyth, G. K. (2010). edgeR: a Bioconductor

package for differential expression analysis of digital gene expression data. *Bioinformatics*,

26(1):139–140.

Sohn, M. B. and Li, H. (2017). A GLM-based latent variable ordination method for microbiome

samples. *Biometrics*, 74(2):448–457.

Warton, D. I., Blanchet, F. G., O’Hara, R. B., Ovaskainen, O., Taskinen, S., Walker, S. C.,

et al. (2015). So many variables: joint modeling in community ecology. *Trends in Ecology*

& *Evolution*, 30(12):766–779.

Weiss, S., Xu, Z. Z., Peddada, S., Amir, A., Bittinger, K., Gonzalez, A., et al. (2017).

Normalization and microbial differential abundance strategies depend upon data characteristics.

*Microbiome*, 5(1):27.

Westling, T. and McCormick, T. (2019). Beyond prediction: A framework for inference with

variational approximations in mixture models. *Journal of Computational and Graphical*

*Statistics*, 28(4):778–789.

Xu, T., Demmer, R. T., and Li, G. (2021). Zero-inflated Poisson factor model with application

to microbiome read counts. *Biometrics*, 77(1):91–101.

Zeileis, A., Kleiber, C., and Jackman, S. (2008). Regression models for count data in R.

*Journal of Statistical Software*, 27(8):1–25.

**2^nd^ round**

**Reviewer 1**

The authors have addressed all my concerns.

**Reviewer 2**

Thanks, my previous review comments have been addressed in the revision.
